# CSTF2 mediated mRNA *N*^6^-methyladenosine modification drives pancreatic ductal adenocarcinoma m^6^A subtypes

**DOI:** 10.1038/s41467-023-41861-y

**Published:** 2023-10-10

**Authors:** Yanfen Zheng, Xingyang Li, Shuang Deng, Hongzhe Zhao, Ying Ye, Shaoping Zhang, Xudong Huang, Ruihong Bai, Lisha Zhuang, Quanbo Zhou, Mei Li, Jiachun Su, Rui Li, Xiaoqiong Bao, Lingxing Zeng, Rufu Chen, Jian Zheng, Dongxin Lin, Chuan He, Jialiang Zhang, Zhixiang Zuo

**Affiliations:** 1 State Key Laboratory of Oncology in South China, Guangdong Provincial Clinical Research Center for Cancer, Sun Yat-sen University Cancer Center, Guangzhou, China; 2 Department of Pancreaticobiliary Surgery, Sun Yat-sen Memorial Hospital, Sun Yat-sen University, Guangzhou, China; 3 Department of Pathology, Sun Yat-sen University Cancer Center, Guangzhou, China; 4 Guangdong Provincial People’s Hospital & Guangdong Academy of Medical Sciences, Guangzhou, China; 5 Jiangsu Key Lab of Cancer Biomarkers, Prevention and Treatment, Collaborative Innovation Center for Cancer Medicine, Nanjing Medical University, Nanjing, China; 6 Affiliated Cancer Hospital and Institute of Guangzhou Medical University, Guangzhou, China; 7 Department of Etiology and Carcinogenesis, National Cancer Center/National Clinical Research Center/Cancer Hospital, Chinese Academy of Medical Sciences and Peking Union Medical College, Beijing, China; 8 Department of Chemistry, The University of Chicago, Chicago, IL USA; 9 Howard Hughes Medical Institute, The University of Chicago, Chicago, IL USA; 10 Department of Biochemistry and Molecular Biology, and Institute for Biophysical Dynamics, The University of Chicago, Chicago, IL USA

**Keywords:** Cancer epigenetics, Cancer epigenetics, Pancreatic cancer

## Abstract

*N*^6^**-**methyladenosine (m^6^A) modification of gene transcripts plays critical roles in cancer. Here we report transcriptomic m^6^A profiling in 98 tissue samples from 65 individuals with pancreatic ductal adenocarcinoma (PDAC). We identify 17,996 m^6^A peaks with 195 hyper-methylated and 93 hypo-methylated in PDAC compared with adjacent normal tissues. The differential m^6^A modifications distinguish two PDAC subtypes with different prognosis outcomes. The formation of the two subtypes is driven by a newly identified m^6^A regulator CSTF2 that co-transcriptionally regulates m^6^A installation through slowing the RNA Pol II elongation rate during gene transcription. We find that most of the CSTF2-regulated m^6^As have positive effects on the RNA level of host genes, and CSTF2-regulated m^6^As are mainly recognized by IGF2BP2, an m^6^A reader that stabilizes mRNAs. These results provide a promising PDAC subtyping strategy and potential therapeutic targets for precision medicine of PDAC.

## Abstract (web summary)

N6-methyladenosine (m6A) modification has important implications in different cancer subtypes. Here, the authors perform transcriptomic m6A profiling to identify two subtypes of pancreatic ductal adenocarcinoma with differential m6A modifications and different clinical outcomes, which is driven by m6A regulator CSTF2.

## Introduction

Pancreatic ductal adenocarcinoma (PDAC), ranking the fourth leading cause of cancer-related death in the world^1^, is often diagnosed at an advanced stage. The improvement in the outcome of PDAC is lagging behind many other malignancies, due to the lack of effective approaches in early diagnosis, treatment, and difficulties for therapeutic agents to access tumor sites^2,3^. Chemotherapy is still the main treatment strategy for most advanced PDAC, though only benefits a subset of patients^4^. Therefore, it is of urgent need to develop superior markers and therapeutic targets based on better understanding of the biology of PDAC.

Molecular subtyping has been used to guide clinical treatment in many cancer types, such as breast cancer and colon cancer, but has yet to be effective in PDAC^5^. Genome-wide association studies and whole-genome sequencing studies on PDAC have provided many potential molecular biomarkers for PDAC subtyping^6,7^. Based on transcriptomic data, several studies have classified PDAC into distinct molecular subtypes^8–10^. Law et al.^11^ have classified PDAC into four subtypes with distinct microenvironment based on proteomic analysis. Although none of these studies have been implicated in clinical practice, these studies suggested that molecular subtyping could be a promising feature in guiding clinical PDAC treatment.

The RNA modifications are a new epigenetics layer of posttranscriptional regulation of genes. *N*^6^-adenosine methylation (m^6^A), as one of the most prevalent RNA modifications, plays an important role in a variety of biological processes, such as cell fate determination^12–14^, circadian clock regulation^15^, adipogenesis^16^, cell cycle arrest, and apoptosis^17^. Accumulating evidence has suggested that the aberrant RNA m^6^A modifications are important events in human cancer development and progression^18–21^. Recently, we have demonstrated that m^6^A modifications stimulated by cigarette smoke can promote excessive miR-25-3p maturation, which enhances pancreatic cancer progression^18^. We hypothesized that m^6^A in RNA might hold great promise as molecular markers for PDAC subtyping.

In the present study, we have performed transcriptome-wide m^6^A-sequencing on ribosomal RNA (rRNA)-depleted RNAs of 98 pancreatic tissue samples from 65 individuals with PDAC. We have identified m^6^A profiles in PDAC distinct from the adjacent normal tissues, based on which defining PDAC subtypes. Further study uncovered CSTF2 as an m^6^A deposition mediator, driving the formation of two PDAC subtypes. Furthermore, we found that the CSTF2-regulated m^6^A methylation program can be recognized mostly by IGF2BP2, the m^6^A stabilized reader, promoting oncogenic pathways, suggesting that the CSTF2-associated PDAC m^6^A subtyping can serve as a promising therapeutic strategy.

## Results

### Transcriptome-wide m^6^A mapping in PDAC

**Fig. 1:**
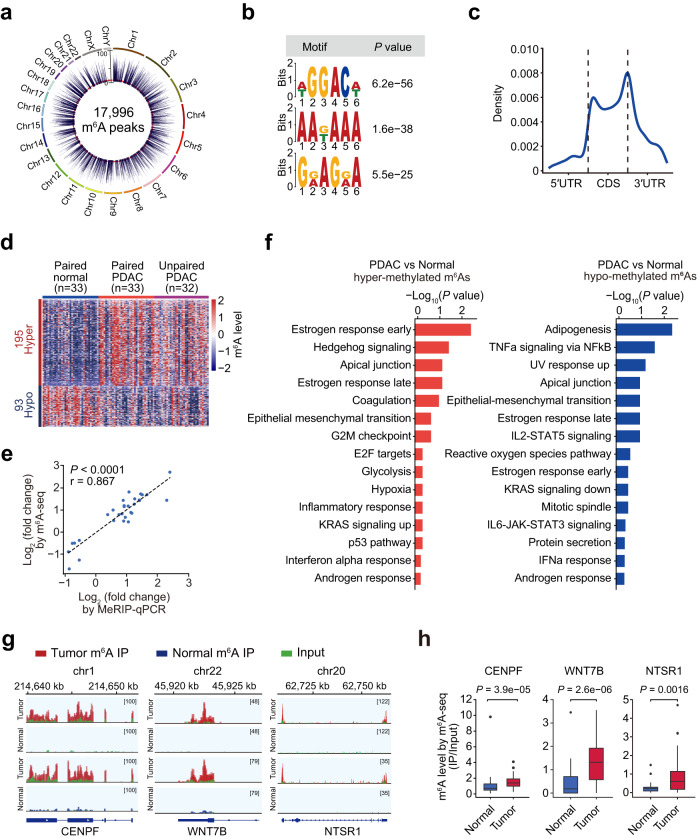
Transcriptome-wide mapping of m^6^A modification in PDAC. **a** Circos plot showing identified RNA m^6^A modifications in 65 PDAC tumor and 33 normal tissue samples. Each bar represents an m^6^A and bar height indicates sample number having the m^6^A. The red dashed circle indicates the cutoff (set as five) for sample number having the m^6^A. **b** Sequence logo representing the enriched sequence motif in m^6^As analyzed by the MEME tool. **c** Location distribution for m^6^As in mRNAs. **d** Heatmap showing different m^6^A levels in 33 paired PDAC tumor and normal samples and 32 unpaired PDAC tumor samples (FDR < 0.1 of paired Wilcoxon test). Each row represents a differentially methylated m^6^A and each column represents a sample. **e** Spearman correlation of fold changes of 29 aberrant m^6^As (tumor/normal) determined by m^6^A-seq or MeRIP-qPCR. **f** Significantly enriched hallmarks for hypermethylated m^6^As (*left panel*) or hypo-methylated m^6^As (right panel) by the Reactome pathway analysis. **g** The abundance of m^6^A in indicated RNAs in tumor tissues and adjacent normal tissues by m^6^A-seq. Number represents the range of the m^6^A signals. **h** The abundance of indicated RNAs that are qualified by m^6^A-seq in tumor tissues (*n* = 33) and adjacent normal tissues (*n* = 33). Boxplots indicate median (middle line), 25th, 75th percentile (box), and 5th and 95th percentile (whiskers). *P* values were from Wilcoxon rank-sum test.

We performed m^6^A-sequencing (m^6^A-seq) on rRNA-depleted RNAs of 98 pancreatic samples from 65 individuals, including 33 pairs of PDAC and corresponding normal tissue and another 32 PDAC samples (Supplementary Table 1 and Supplementary Data 1), and identified 26,684 m^6^A peaks by using MACS2^22^ and MeTPeak^23^. After removing 462 (1.7%) peaks at the “A” of the transcription start site (TSS) and BCA motifs, which may be *N*^6^,2’-*O*-dimethyladenosine (m^6^Am) that can also be captured by the m^6^A antibody^24^, and those m^6^As that were not detected in at least 5 different samples, 17,996 m^6^A peaks were used in further analysis (Fig. 1a and Supplementary Data 2). Among these m^6^A peaks, 15,708 (87.3%) have been recorded in the RMBASE^25^ with many transcripts that are well-known to be m^6^A-modified (Supplementary Fig. 1a). Moreover, the identified m^6^A sites were enriched in the classical GGACH motif (Fig. 1b) and the regions near the start- and stop-codons (Fig. 1c). These results are in line with previous findings^26,27^.

These 17,996 m^6^A peaks were mainly located in genetic regions coding for messenger RNAs (mRNAs, 95.9%) (Supplementary Fig. 1b), consistent with results from polyA^+^ RNA m^6^A-seq^28^. However, because we employed rRNA-depleted RNA m^6^A-seq, there were 24.5% of the identified m^6^A peak located in the intron regions which show a proportion of GGACH motif comparable to the exon regions with m^6^A (Supplementary Fig. 1c), and the m^6^As-modified intron regions were usually close to the splicing sites (Supplementary Fig. 1d), implying that the intron regions can also be modified by m^6^A, which may alter mRNA splicing as suggested previously^16,29^.

### Distinguishing two PDAC subtypes by differential m^6^A modifications

Among the 17,996 m^6^A sites, 195 were hypermethylated while 93 were hypo-methylated in 33 tumors compared with those in 33 paired normal tissues (Supplementary Data 3). Most of these differentially methylated m^6^As (265/288) were validated by PDACs by comparing an independent dataset with 32 unpaired PDACs to the 33 normal pancreatic tissues (Fig. 1d). Permutation analysis of tumor and normal sample labels (1000 times) yielded an average of 17 differential m^6^A sites that were far less than observed 288 differential m^6^A sites (Supplementary Fig. 1e), indicating that the identified aberrant m^6^A sites are not random. 96.6% (28/29) of the randomly selected aberrant m^6^A sites were validated by MeRIP-qPCR (Supplementary Table 2), supporting the reliability of our m^6^A-seq data (*r* = 0.867, *P* < 0.0001; Fig. 1e). Moreover, by using the RADAR program^30^, a recently developed analytical tool for detecting differentially methylated loci in MeRIP-seq data, we found that most differentially methylated m^6^As (175/288) were also identified by RADAR, indicating the high confidence of our results.

Most of the differential m^6^A sites are within mRNAs and are enriched around the stop-codons and in 3’UTR regions (Supplementary Fig. 1f). The 288 dysregulated m^6^A sites were enriched in genes related to cancer pathways such as cell cycle and epithelial-mesenchymal transition (Fig. 1f). For example, previously reported oncogenes such as *CENPF*^31,32^, *WNT7B*^33–35^ and *NTSR1*^36,37^ were found to be hyper-m^6^A methylated in tumor versus adjacent normal tissues (Fig. 1g, h).

**Fig. 2:**
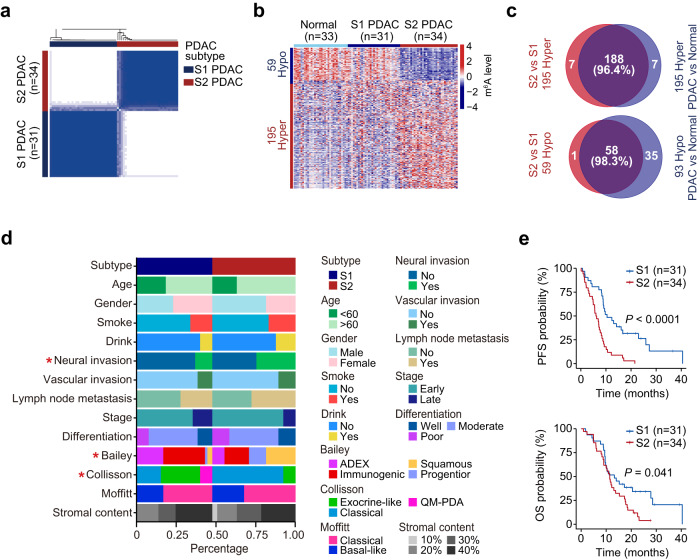
Subtyping of PDAC by transcriptome-wide m^6^A modifications. **a** Discrimination of PDAC as S1 or S2 subtypes by differential m^6^A. **b** Heatmap showing significantly different (FDR < 0.1 of Wilcoxon rank-sum test) m^6^A in the normal, S1 and S2 PDAC tissues. **c** Venn plot showing great overlap between the hyper (upper panel) or hypo-methylated m^6^A (lower panel) in the S2 PDAC subtype versus the S1 PDAC subtype and those in PDAC tumor versus normal. **d** The associations between the m^6^A-defined PDAC subtypes and clinical (sex, age, smoking status, drinking status, tumor stage, differentiation, neural invasion, vascular invasion, and lymph node metastasis) or molecular features (the transcriptional subtype). **e** Kaplan–Meier estimates of progression-free survival (PFS, upper panel) and overall survival (OS, lower panel) in PDAC patients with different m^6^A subtype. *, *P* < 0.05 of Fisher’s exact test in **d**.

Unsupervised consensus clustering of the PDAC patients according to these differential m^6^A peaks further characterizes two PDAC subtypes (designated as S1 and S2, respectively, Fig. 2a). The S2 PDAC showed an m^6^A pattern that was distinct from the S1 PDAC (Fig. 2b), but not different in the adjacent normal tissues of two PDAC subtypes (Supplementary Fig. 2a), suggesting that the subtype patterns are tumor-specific. Moreover, the differentially methylated m^6^As between the S1 and S2 PDAC samples showed no difference between S1 PDAC samples and adjacent normal tissue samples (Fig. 2b), and had a large overlap with the differentially methylated m^6^As between adjacent normal and tumor tissues (Fig. 2c), indicating a S2 PDAC-specific m^6^A dysregulation. The m^6^As of the genes in cancer pathways such as cell cycle and epithelial-mesenchymal transition were hypermethylated in S2 PDAC samples compared to S1 PDAC samples (Supplementary Fig. 2b). For instances, differentially methylated m^6^As in genes such a*s CENPF*, *WNT7B* and *NTSR1* between tumor and normal tissues were hypermethylated in S2 PDAC samples compared to S1 PDAC samples (Supplementary Fig. 2c, d).

We analyzed the correlations of the two subtypes with known clinical factors such as sex, age, smoking status, drinking status, tumor stage, differentiation, vascular invasion, and lymph node metastasis. The results were all negative except for neural invasion (Fig. 2d). The stromal content is not significantly different between the two subtypes in tumor tissues used for m^6^A sequencing (Fig. 2d and Supplementary Table 1), indicating that these subtype patterns are PDAC intrinsic features. We further examined whether the m^6^A subtypes of PDAC are correlated with previously reported transcriptional subtypes^8–10^. We found that the frequencies of Bailey’s squamous subtype and Collisson’s classical subtype were significantly higher in S2 PDAC than in S1 PDAC (Fig. 2d).

Survival analysis revealed that the S2 PDAC had a median progress-free survival (PFS) time and overall survival (OS) time that were significantly shorter than the S1 PDAC (6.6 versus 11.2 months, log-rank *P* < 0.0001 and 11.4 versus 13.3 months, log-rank *P* = 0.041, respectively; Fig. 2e), with the HRs being 4.28 (95% CI = 1.53−11.96) and 3.31 (95% CI = 1.12−8.78), respectively, adjusted for clinical features, mutations of *KRAS*/*TP53* and transcriptional subtypes reported previously^8,10^. However, we did not find a significant association between survival and known transcriptional subtypes (Supplementary Fig. 2e), suggesting our m^6^A subtypes are independent of other transcriptional subtypes. Interestingly, we found that the T-cell and B-cell markers were comparatively lower in S2 subtype than S1 subtype (Supplementary Fig. 2f), suggesting a different immune phenotype between the two subtypes.

### CSTF2 drives the PDAC m6A subtype formation

**Fig. 3:**
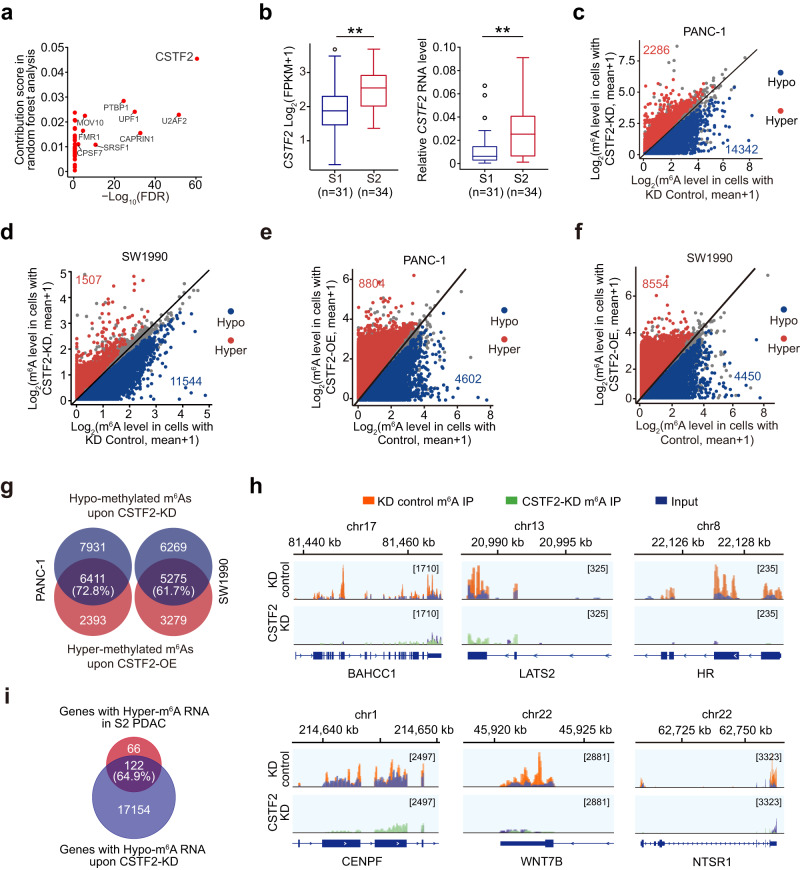
CSTF2 is a key protein to promote mRNA m^6^A deposition in S2 PDAC subtype. **a** Spearman correlation between the expression of RNA binding proteins (RBPs) and hypermethylated m^6^As in the S2 PDAC subtype. The *x* axis represents log_10_(FDR) in analyzing the enrichment of the RBP-correlated hypermethylated m^6^As against background. The *y* axis represents the contribution score of each RBP to the hypermethylated m^6^A based on random forest analysis. **b** CSTF2 expression levels in two PDAC subtypes determined by RNA sequencing (left panel) or qRT-PCR (right panel). The line in the middle of the box is plotted at the median while the upper and lower hinges indicated 25th and 75th percentiles. **P < 0.01 of Wilcoxon rank-sum test. **c** The effect of *CSTF2* on m^6^A levels in PANC-1 cells. There were 2286 hypermethylated m^6^As (red) and 14,342 hypo-methylated m^6^As (blue) in cells with *CSTF2* knockdown (KD). **d** The effect of *CSTF2* on m^6^A level in SW1990 cells. There were 1507 hypermethylated m^6^As (red) and 11,544 hypo-methylated m^6^As (blue) in cells with *CSTF2* knockdown (KD). **e**, **f** Scatter plot of m^6^A levels in PANC-1 cells (**e**) and SW1990 cells (**f**) with or without forced *CSTF2* expression. There were 8804 hypermethylated m^6^As (red) and 4602 hypo-methylated m^6^As (blue) in cells with *CSTF2* overexpression in PANC-1 cells. There were 8554 hypermethylated m^6^As (red) and 4450 hypo-methylated m^6^As (blue) in cells with *CSTF2* overexpression in SW1990 cells. **g** Venn plot showing overlap between hypo-methylated m^6^As in cells with *CSTF2* knockdown and hypermethylated m^6^As in cells with *CSTF2* overexpression. **h** Integrative genomics viewer (IGV) plots show different abundance of m^6^A in the depicted transcripts between PDAC cells with or without *CSTF2* KD. **i** Venn plot showing the mRNAs with hyper m^6^As in the S2 PDAC subtype and mRNA with hypo m^6^As in PDAC cells with *CSTF2* KD. Cut off for significantly differential m^6^A methylation are defined as │fold change│>1.2 in **c**−**f**.

We next explored the mechanism underlying the formation of PDAC subtypes. First, we applied random forest analysis and spearman correlation analysis to examine the correlation of hypermethylated m^6^A in the S2 PDAC with RNA binding proteins (RBPs) that have the binding sites supported by CLIP sequencing data in POSTAR2 database^38^ overlapped with the m^6^A peaks or with known m^6^A writers and erasers (Supplementary Fig. 3a). We found that *Cleavage Stimulation Factor 2* (*CSTF2*) RNA levels were most significantly correlated with the levels of hypermethylated m^6^A sites in S2 PDAC (Fig. 3a). Both *CSTF2* RNA and protein level were significantly higher in PDAC than in adjacent normal tissues (Supplementary Fig. 3b−d) and in the S2 PDAC than the S1 PDAC (Fig. 3b and Supplementary Fig. 3e), while another two candidates, U2AF2 and CAPRIN1, showed little difference on RNA levels between the S2 and S1 PDAC (Supplementary Fig. 3f). PDAC cell lines (PANC-1 and SW1990) with moderate expression level of *CSTF2* were chosen for experiments subsequently (Supplementary Fig. 3g). We found that when the *CSTF2* was knocked down in PANC-1 and SW1990 cells, methylation levels were substantially decreased in 86% (14,342/16,628) and 88% (11,544/13,051) of differential m^6^A sites, respectively (Fig. 3c, d and Supplementary Fig. 3h). The effect of CSTF2 on m^6^A were further verified by m^6^A-LC-MS (Supplementary Fig. 3i) and m^6^A-ELISA (Supplementary Fig. 3j), while knockdown of *U2AF2* and *CAPRIN1* showed minute effect of global m^6^A level (Supplementary Fig. 3k−m). Moreover, when *CSTF2* was ectopically overexpressed in the same cell lines, 8804 and 8554 of m^6^A sites were hypermethylated (Fig. 3e, f), with 72.8% (6411/8804) and 61.7% (5275/8854) overlapping of hypo-methylated m^6^A in the two examined cell types with *CSTF2* knockdown (Fig. 3g). Significantly dysregulated m^6^As (Fig. 3h) upon *CSTF2*-knockdown were further verified by MeRIP-qPCR (Supplementary Fig. 3n), which could be rescued by forced-expressed *CSTF2* (Supplementary Fig. 3o, p), but not affected by *U2AF2* or *CAPRIN1* knockdown (Supplementary Fig. 3q). Moreover, 64.9% (122/188) hypermethylated in the S2 PDAC are hypo-methylated in cells with *CSTF2* knockdown (Fig. 3i). Together, these results suggest that CSTF2 may regulate mRNA m^6^A formation in PDAC.

### CSTF2 promotes the malignant phenotypes of PDAC cells

**Fig. 4:**
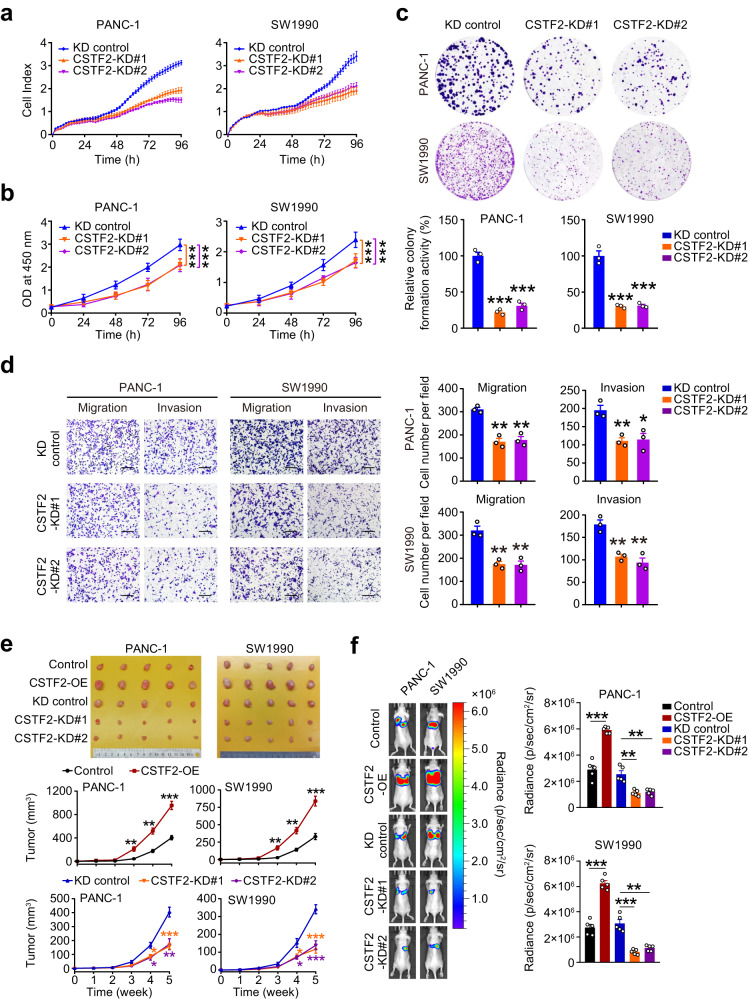
Knockdown of CSTF2 inhibits proliferation and metastasis of PDAC cells. **a**, **b** *CSTF2* KD repressed PDAC cell proliferation gauged by electrical impedance expressed as decreasing cell index (**a**) or measured by CCK-8 assays (**b**). Data of **a**, **b** are means ± S.D. (*n* = 3). **c** *CSTF2* KD inhibited PDAC cell colony formation. Upper panels are representative pictures of colony formation; lower panels show quantitative statistics of relative colony formation activity (means ± S.E.M., *n* = 3). **d** *CSTF2* KD repressed PDAC cell migration and invasion determined by transwell assays. Upper panels are representative pictures showing different abilities of cell migration and invasion; Lower panels show quantitative statistics of migration and invasion abilities. Data are means ± S.E.M. in (*n* = 3) of three independent experiments. Scale bars, 200 μm. **e** Effects of *CSTF2* on the growth of xenograft tumors derived from PDAC cells in vivo in nude mice. Shown were the subcutaneous xenografts obtained at the end of experiments (left panel) and the curves of xenograft growth (right panel). **f** Effects of *CSTF2* on lung localization of PDAC cells in nude mice (*n* = 5) with injection of cells via tail-vein. Left panels show representative bioluminescence imaging at day 42 after injection of cells, and right panel shows quantitative luminal intensities. Data represent means ± S.E.M. from five mice of each group. Data of **a**−**d** were from three independent experiments. *, *P* < 0.05; **, *P* < 0.01 and ***, *P* < 0.001 of Student’s *t* tests compared with each control.

We then explored the effects of CSTF2 on malignant phenotypes of PDAC cells. In vitro experiments showed that the knockdown of *CSTF2* substantially suppressed the abilities of cell proliferation, colony formation, cell cycle, migration, and invasion of PDAC cells (Fig. 4a−d, Supplementary Fig. 4a−c). By using mouse subcutaneous xenograft models, we also found that *CSTF2* overexpression significantly enhanced but silence markedly suppressed the growth rates of PDAC tumor (Fig. 4e). Furthermore, forced expression of *CSTF2* promoted lung metastasis of PDAC cells while *CSTF2* knockdown showed opposite effects (Fig. 4f). Additionally, the *CSTF2* knockdown induced inhibition of malignant phenotypes can be rescued by forced-expressed *CSTF2*, implying the on-target effect of *CSTF2* knockdown (Supplementary Fig. 4d−f). Notably, the malignant phenotypes promoted by forced-expressed *CSTF2* could be partially alleviated by knockdown of *CENPF*, *WNT7B,* or *NTSR1* (Supplementary Fig. 4g, h), implying that CSTF2 may function via modulating m^6^A of specific genes.

### CSTF2 mediates m^6^A deposition by retarding elongation

We next investigated how CSTF2 mediated m^6^A deposition. We found that neither the expressions nor the subcellular localizations of the known m^6^A writers or erasers were affected by *CSTF2* knockdown in PDAC cells (Supplementary Fig. 5a−c). The intact methyltransferase complex was not affected by *CSTF2* knockdown in PDAC cells (Supplementary Fig. 5d). Depletion of *CSTF2* has relatively small effect on global APA profiling (Supplementary Fig. 5e), which is similar with previous studies reporting that CSTF2T plays a redundant role in regulating APA with CSTF2T could be upregulated upon *CSTF2* knockdown (Supplementary Fig. 5f) and only co-depletion of *CSTF2* and *CSTF2T* leads to obvious APA changes^39,40^. Moreover, genes with significant APA changes hold little overlap with hypo-methylated genes upon *CSTF2* knockdown (38/7426). The results above indicate that the phenotypes observed upon *CSTF2* knockdown were unlikely mediated through APA.

**Fig. 5:**
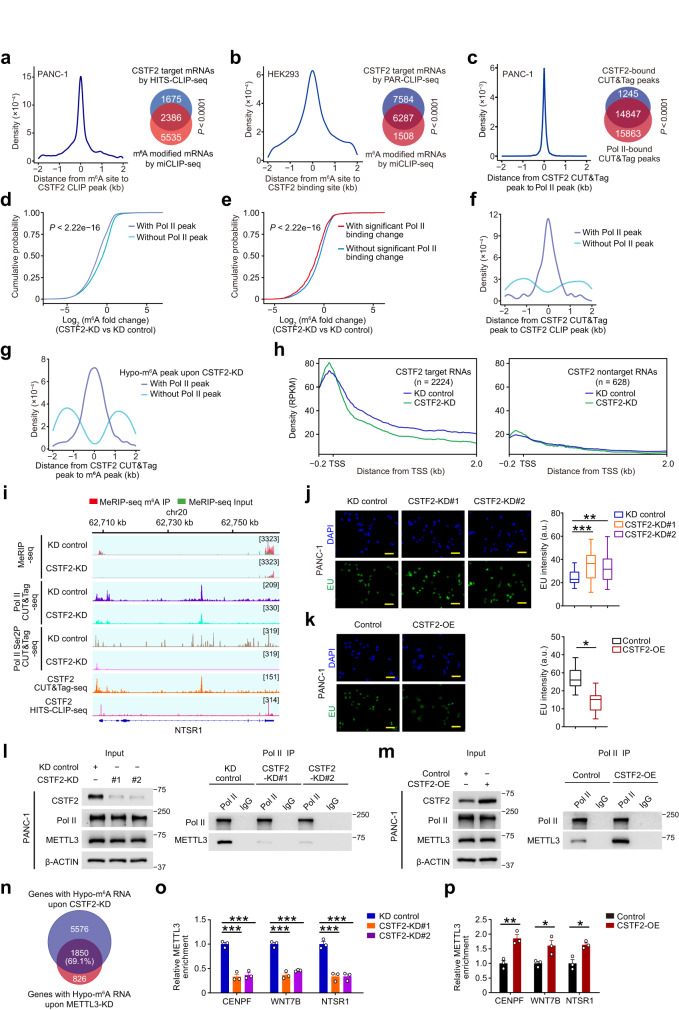
CSTF2 mediates m^6^A deposition by retarding elongation. **a**, **b** Co-localization of the CSTF2 binding sites and the m^6^A sites in RNA in PANC-1 (**a**) or HEK293 cells (**b**). The line plot shows distance between the CSTF2 binding sites (*left panel*) and the m^6^A sites in RNA within 2-kb region and the Venn plots show the corresponding overlapping proportion (*right panel*). *P* value for Fisher’s exact test. **c** Co-localization of the DNA binding sites of CSTF2 and RNA Pol II in PANC-1 cells. **d**, **e** Genes enriched with Pol II binding (**d**) or with significant Pol II binding change (**e**) experienced more dramatic m^6^A change upon *CSTF2*-KD. *P* value of **d**, **e** were from Wilcoxon rank-sum test. **f** Co-localization of the DNA and RNA-binding sites of CSTF2 in PANC-1 cells. Purple line and blue line represents those CSTF2 CLIP peak overlapped (purple) or not overlapped (blue) with Pol II CUT&Tag peak, respectively. **g** Co-localization of DNA-binding sites of CSTF2 and RNA m^6^A sites in PANC-1 cells. Purple line and blue line represent those CSTF2-targeted m^6^A peak overlapped (purple) or not overlapped (blue) with Pol II CUT&Tag peak, respectively. **h** Comparison of the RNA Pol II density along the CSTF2 target mRNA (*left*: CSTF2 target RNAs; *right*: CSTF2 non-target RNAs) in PANC-1 cells upon *CSTF2*-KD. **i** Shown are representative tracks of transcript experiencing m^6^A level and Pol II binding densities change upon *CSTF2* KD. **j**, **k** Representative images of 5-ethynyluridine (EU) labeling in PANC-1 cells (left panel) showing the effect of *CSTF2* KD (**j**) or overexpression (**k**) of three independent experiments, and quantification analysis of EU signals from one representative experiment (right panel). Scale bar, 100 μm. Boxplots indicate median (middle line), 25th, 75th percentile (box) and 5th and 95th percentile (whiskers) (Cell number: *n* = 27, 31 and 29 for KD control, CSTF2-KD#1 and CSTF2-KD#2; *n* = 29 and 24 for Control and CSTF2 OE, respectively) **l**, **m** Showing the effects of *CSTF2* KD (**l**) or overexpression (**m**) on the interaction between Pol II and METTL3 in PANC-1 cells. **n** Showing the overlap between mRNAs with hypo-m^6^As upon *CSTF2* KD and *METTL3* KD. **o**, **p** CLIP-qPCR showed that *CSTF2* KD impaired (**o**) but overexpression enhanced (**p**) METTL3 binding to target transcripts in PANC-1. Data are the mean ± S.E.M. of three independent experiments in **o**, **p**. *P < 0.05; **P < 0.01 and ***P < 0.001 of Student’s *t*-test.

Our CLIP sequencing data showed that the CSTF2 RNA binding sites are well overlapped with m^6^A sites in RNA (Fig. 5a and Supplementary Fig. 5g−j), consistent with reported public CLIP sequencing data (Fig. 5b). Previous studies reported that CSTF2 can directly interact with RNA polymerase II (RNA Pol II)^41,42^ that is known to recruit the m^6^A methyltransferase complex (MTC) co-transcriptionally^43^, suggesting that CSTF2 might affect m^6^A deposition through MTC and RNA Pol II. We thus performed CUT&Tag sequencing of CSTF2 and RNA Pol II, and the results showed a good overlap of genomic binding positions of CSTF2 and RNA Pol II (Fig. 5c). We observed that m^6^A peaks with Pol II occupancy showed a significantly greater reduction in m^6^A levels upon *CSTF2* knockdown than those peaks without Pol II occupancy (Fig. 5d). Furthermore, we found that genes exhibiting substantial changes in Pol II occupancy also displayed a greater reduction in m6A levels upon *CSTF2* knockdown (Fig. 5e). These results suggest that m^6^A sites whose formation is more reliant on Pol II may be particularly vulnerable to the effects of *CSTF2* knockdown. Moreover, we found that CSTF2-binding sites in DNA were co-localized with CSTF2 binding sites and m^6^A sites in RNA, and the co-localization was associated with RNA Pol II (Fig. 5f, g). These results implied that RNA Pol II may indeed play a role in mediating m^6^A depositions regulated by CSTF2.

Previous study reported that CSTF2 may function as a rate-limiting factor in the elongation of RNA Pol II^44^, and a recent study showed that prolonged elongation rate may help RNA Pol II to recruit m^6^A writer METTL3^43^. We further performed CUT&Tag sequencing of Pol II and Pol II-Ser2P upon *CSTF2* knockdown. Significant decreases in RNA Pol II and Pol II-Ser2P density were observed in those genes with hypomethylated-m^6^A upon *CSTF2* knockdown, while the RNA Pol II density of CSTF2 non-targets was not affected (Fig. 5h and Supplementary Fig. 5k). We also observed a slight increase in H3K79me2 and H3K36me3, but no significant changes in the enrichment of Pol II-ser5P (Supplementary Fig. 5k). This was illustrated by the representative genomic tracks of CSTF2 targets such as *CENPF*, *WNT7B* and *NTSR1* genes **(**Fig. 5i and Supplementary Fig. 5l). These findings are in line with the fact that faster elongation leads to lower RNA Pol II density in the gene body^45^. Moreover, using 5,6-dichlorobenzimidazole 1-beta-D-ribofuranoside (DRB) in combination with global nuclear run-on followed by sequencing (GRO-seq) (Supplementary Fig. 5m), we found that knockdown of *CSTF2* moderately increased the elongation rate of target RNAs (Supplementary Fig. 5n), which was validated by elongation rate experiments subsequently (Supplementary Fig. 6a−c). We also found that *CSTF2* knockdown facilitated but ectopic overexpression of *CSTF2* attenuated the synthesis of nascent RNA in PDAC cells (Fig. 5j, k and Supplementary Fig. 6d, e), confirming that CSTF2 action decreased the elongation rate of Pol II. We therefore hypothesized that CSTF2 might facilitate the METTL3 recruitment via prolonging elongation rate of RNA Pol II, thus promoting m^6^A deposition. We found that forced *CSTF2* expression changes in PDAC cells resulted in substantial variations of the RNA Pol II and METTL3 interaction (Fig. 5l, m and Supplementary Fig. 7a, b). Similar results were also observed between the activated elongating form of Pol II, phosphorylation of the C-terminal domain (Pol II-Ser2P) and METTL3 upon forced CSTF2 expression changes (Supplementary Fig. 7c, d), whereas global Pol II-Ser2P was not affected, suggesting that CSTF2-retarded Pol II recruited more METTL3.

Notably, knockdown of *CSTF2* caused comparable decrease of global m^6^A level with that by *METTL3* knockdown (Supplementary Fig. 7e, f), and the hypomethylated-m^6^A in cells with *METTL3* knockdown overlapped with 69% (1850 of 2676) of m^6^A produced by CSTF2 (Fig. 5n). *CSTF2* knockdown caused less METTL3 binding around the m^6^A region of the target transcript but ectopic overexpression of *CSTF2* strengthened the interaction (Fig. 5o, p and Supplementary Fig. 7g). It is reported that CSTF2 effects on the binding of elongation factors on transcripts co-transcriptionally^44^. Notably, we found that knocked down or inhibited the elongation factors AFF1/4 in HEK293T cells markedly attenuated the elongation rate of CSTF2 target genes, while effects on CSTF2 non-target genes tended to be weaker (Supplementary Fig. 7h−j). *CSTF2* knockdown in PDAC cells promoted the recruitment of AFF1/4, ensuring efficient elongation but ectopic overexpression of *CSTF2* attenuated the recruitment, leading to slowing elongation (Supplementary Fig. 7k, l). These results strongly support that CSTF2 promotes m^6^A depositions by slowing down the Pol II elongation, therefore facilitating the recruitment of METTL3 co-transcriptionally.

### CSTF2-regulated m^6^As enhance RNA stability

**Fig. 6:**
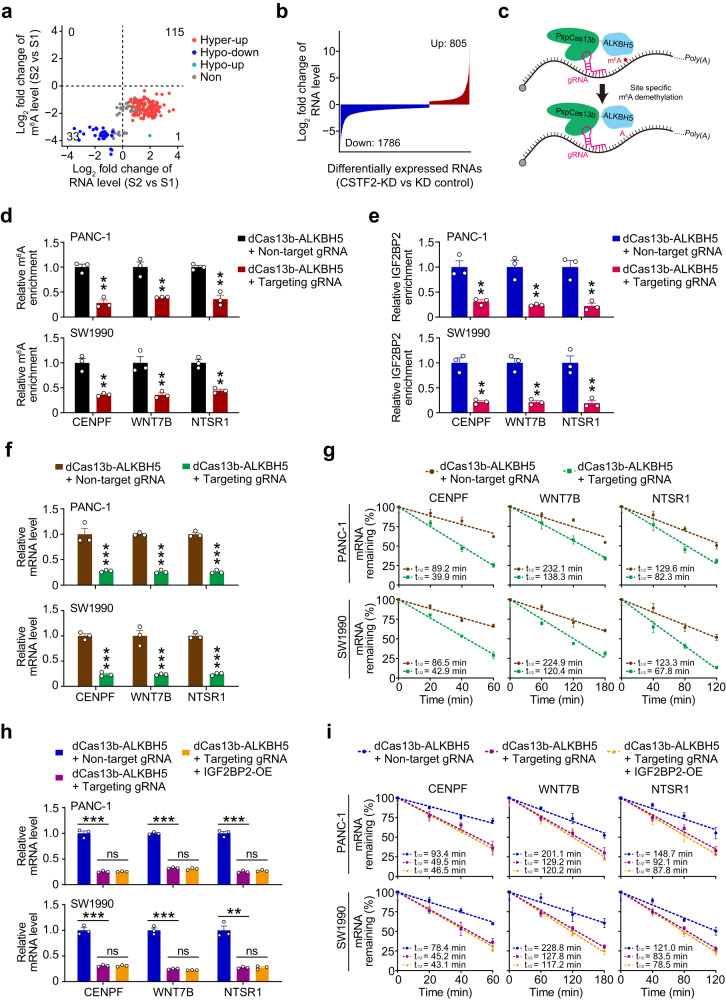
Aberrant m^6^A enhances mRNA stability. **a** Correlations of levels of m^6^As with levels of their host RNAs. Hyper-up, increase in levels of both m^6^As and their host RNAs in S2 PDAC versus S1 PDAC; hypo-down, decrease in levels of both m^6^As and their host RNA levels in S2 PDAC versus S1 PDAC; hypo-up, decrease in m^6^A level but increase in RNA level in S2 PDAC versus S1 PDAC; Non-significant change, levels of RNA did not significantly change in S2 PDAC versus S1 PDAC. **b** Waterfall plot of differentially expressed CSTF2 target RNAs upon *CSTF2* knockdown in PANC-1 cell. **c** Diagram of dCas13 based m^6^A editing system. **d** Relative m^6^A enrichment of *CENPF*, *WNT7B*, *NTSR1* transcript detected by MeRIP-qPCR upon transfected with dCas13b-ALKBH5 and non-target gRNA or gRNA targeting m^6^A of specific RNAs, respectively. **e** Relative IGF2BP2 enrichment of indicated transcript detected by CLIP-qPCR upon transfected with dCas13b-ALKBH5 and non-target gRNA or gRNA targeting m^6^A of specific RNA, respectively. **f**, **g** Relative mRNA level (**f**) and half-lives (**g**) of *CENPF*, *WNT7B*, *NTSR1* detected by qRT-PCR upon transfected with dCas13b-ALKBH5 and non-target gRNA or gRNA targeting m^6^A of indicated RNA, respectively. **h**, **i** Relative mRNA level (**h**) and half-lives (**i**) of *CENPF*, *WNT7B*, *NTSR1* detected by qRT-PCR upon manipulating the m^6^A site by dCas13 based m^6^A editing system with or without rescue with forced-expressed IGF2BP2. Data are the mean ± S.E.M. of three independent experiments in **d**−**i**. **P < 0.01 and ***P < 0.001 of Student’s *t* test. ns, not significant.

We then explored the effects of m^6^As on their host RNA levels in PDAC and found that 205 m^6^As (148 RNAs) out of the 254 differentially methylated m^6^As between S1 and S2 PDAC subtype had impacts on their host RNA levels (Fig. 6a and Supplementary Data 4). For example, both the m^6^A level and RNA level of some genes in cancer-related pathways such as cell cycle and epithelial-mesenchymal transition were significantly different between S1 and S2 (Supplementary Fig. 8a). Among the 148 RNAs, the m^6^A levels and RNA levels of 115 RNAs were both upregulated in S2 PDAC compared to S1 PDAC, while the m^6^A levels and RNA levels of 33 RNAs were both downregulated (Fig. 6a). We also found higher RNA levels of CSTF2 target genes in PDAC tissues comparing with that in normal tissues (Supplementary Fig. 8b). The positive correlation between m^6^A level and RNA level was also observed in PDAC cells, where RNA level downregulation of many RNAs (805/5222) upon *CSTF2* knockdown will show hypomethylation of m^6^A levels, but only few RNAs (9/5222) will show lengthened 3’UTR (Fig. 6b and Supplementary Fig. 8c), indicating that the CSTF2-regulated m^6^As but not CSTF2-regulated APAs may contribute to the increased RNA levels. IGF2BP family is reported to be an m^6^A reader stabilized transcripts^46^. Since the expression of IGF2BP2 was positively correlated with most of the 254 differentially methylated m^6^As between S1 and S2 PDAC subtype (Supplementary Fig. 8d), we next investigated the role of IGF2BP2 in the CSTF2-regulated m^6^As. We found that the expression of IGF2BP2 was not altered by *CSTF2* knockdown (Supplementary Fig. 8e). However, both *CSTF2* knockdown and *IGF2BP2* knockdown caused similar changes in expression levels of CENPF, WNT7B, NTSR1 (Supplementary Fig. 8f−h). *CSTF2* knockdown dampened the binding of IGF2BP2 to the m^6^A region of the target transcript (Supplementary Fig. 8i), without altering the binding enrichment of YTHDF1/2/3 on these RNAs (Supplementary Fig. 8j). The effect of *CSTF2* knockdown on transcripts can be rescued by ectopic expression of CSTF2 implying that the effect is on-target (Supplementary Fig. 8k). Both *CSTF2* knockdown and *IGF2BP2* knockdown caused similar change on stabilities of CENPF, WNT7B, NTSR1 transcripts (Supplementary Fig. 8l). Furthermore, we conducted dCas13 based m^6^A editing and gRNA to specifically manipulate the m^6^A site (Fig. 6c and Supplementary Fig. 9a). The downregulation of m^6^A level were verified (Fig. 6d) which dampened the binding of IGF2BP2 (Fig. 6e), leading to downregulation of both the mRNA levels (Fig. 6f) and half-lives of transcripts (Fig. 6g), specifically (Supplementary Fig. 9b−g). Forced-expressed IGF2BP2 failed to rescue the effect of downregulation of m^6^A level on both the mRNA levels and half-lives of transcripts (Fig. 6h, i and Supplementary Fig. 9h). Taken together, the above results demonstrated that CSTF2-regulated m^6^As enhance the RNA stability via IGF2BP2.

## Discussion

Increasing evidence has shown that m^6^A modifications are important in the pathogenesis of various types of cancer^20,21,47–49^. However, their global function and regulation in cancer are still largely unknown, mainly due to the lack of m^6^A-seq data from larger sample sizes of cancer patients. In the present study, we have performed a transcriptome-wide m^6^A-seq and mapping of m^6^A modifications in a large set of PDAC samples from 65 patients. To the best of our knowledge, this is the most comprehensive study on mRNA m^6^A methylome landscape in PDAC to date. We have demonstrated that PDAC has obviously different mRNA m^6^A modification compared with adjacent normal tissues, with 68% of m^6^A sites being hypermethylated and 32% of m^6^A sites being hypo-methylated.

Recent high-throughput sequencing studies have revealed a great diversity of PDAC at multi-omics levels, such as genomics, transcriptomics, proteomics, and epigenomics^8–11,50,51^. However, the current data is far more than enough to reveal the complex mechanism underlying the heterogenous disease, let alone guide the clinical treatment based on molecular subtyping in PDAC. In this study, we have innovatively defined two PDAC subtypes using distinct m^6^A modification profiling, which is related to patients’ survival, offering alternative insight into PDAC and informing the development of superior markers or therapeutic regimens based on this finding.

Another important finding is the discovery of CSTF2 as an m^6^A deposition mediator that regulates mRNA m^6^A modification. We have demonstrated that the depletion of CSTF2 in PDAC cells substantially reduced global m^6^A levels but did not change the expression levels of the m^6^A writers and erasers. CSTF2 is well known as a member of the cleavage stimulation factor complex regulating the 3’ end cleavage and alternative polyadenylation (APA)^52^. Our data showed that the knockdown of *CSTF2* alone has a limited effect on global APA, consistent with a previous study, as CSTF2T, the paralog of CSTF2 functioning as an APA regulator similarly, can be upregulated accompanied with *CSTF2* knockdown^39,40^. Pol II termination defect is only observed when *CSTF2* and *CSTF2T* are co-depleted, but not in *CSTF2*-knockdown cells^53^. These data suggest that it is unlikely that the effect of *CSTF2* knockdown on m^6^A levels may be due to its APA-modulating effect. Furthermore, CLIP sequencing shows that CSTF2 binding sites enrich significantly around the m^6^A sites, suggesting that CSTF2 influences m^6^A modifications in a m^6^A site-dependent manner. Recent studies have proposed that the mRNA m^6^A modification is a co-transcriptional process depending on slowing or pausing of transcribing RNA Pol II^29,43^ and CSTF complex can directly interact with RNA Pol II and slow down its elongation rate during Pol II elongation^41,42,44^. Our results together with previous findings indicate that the CSTF2 effect on m^6^A deposition is likely through the mechanism of slowing RNA Pol II elongation rate.

**Fig. 7:**
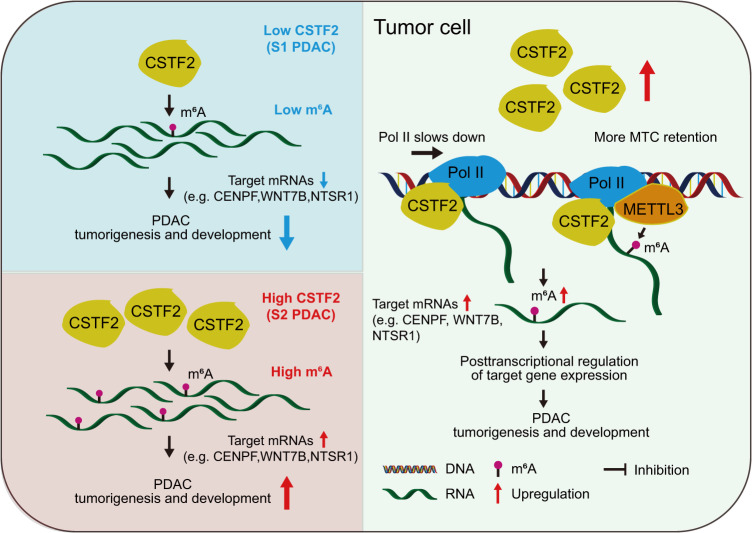
Proposed action model for CSTF2 in RNA m^6^A deposition and formation of PDAC subtypes. High expression of *CSTF2* in PDAC, an m^6^A deposition mediator, causes the aberrant RNA m^6^A which drives the formation of PDAC subtypes by a mechanism of slowing down the transcriptional elongation rate and retention of more methyltransferase complex (MTC). Pancreatic ductal adenocarcinoma (PDAC) can be classified into two subtypes namely subtype 1 (S1) and subtype 2 (S2) based on aberrant m^6^A modifications. Compared with S1, S2 is characterized by high expression of *CSTF2* and high m^6^A level which leads to activation of specific tumor-related pathways.

In conclusion, we have comprehensively deciphered the landscape of transcriptome-wide m^6^A mRNA modification in PDAC. We have identified CSTF2 promoting the m^6^A modification in mRNAs and IGF2BP2 enhancing the stability of mRNAs with hypermethylated m^6^As, which forms a CSTF2-m^6^A-IGF2BP2 axis (Fig. 7). The aberrancy of this m^6^A modification-related axis may contribute to the development and progression of PDAC and thus has the potential clinical applications in PDAC precision medicine.

## Methods

### Patients and tissue specimens

This study was performed according to the Declaration of Helsinki and approved by the Institutional Review Board of Sun Yat-sen University. Written informed consent was obtained from each participant, and all data were anonymously analyzed.

For high-throughput m^6^A-sequencing and disease-relevant molecule analyzing, 65 patients with PDAC were recruited and the distributions of select characteristics are shown in Supplementary Table 1. All patients were recruited at Sun Yat-sen University Sun Yat-sen Memorial Hospital (Guangzhou, China) between 2010 and 2018 and they underwent pancreatectomy and received no treatment before surgery. The diagnosis of PDAC was histopathologically confirmed and tumor stage was classified according to the 7th edition of AJCC Cancer Staging System^54^. The PDAC tumor and non-tumor tissue (≥5 cm away from tumor) samples were collected at surgery from each patient and immediately placed in liquid nitrogen.

### Tissue RNA isolation

Total RNA was isolated from tumor and normal samples with TRIzol reagent (Invitrogen). The tumor and stromal contents were evaluated from the continuous tissue section slides stained with H&E by three board-certified pathologists who were blinded to the patients’ clinicopathological status and only the samples containing ≥60% tumor cells were used. The resultant RNA samples were quantified by measuring absorbance at 260 nm with a UV spectrophotometer and then determined via the RNA6000 Nano assay (Agilent) for an RNA Integrity Number (RIN), and only the samples with RIN ≥ 7.0 were included for further analysis.

### High-throughput m^6^A-sequencing

Total RNA from tissue was digested with DNase I and then subjected to RiboMinus (Illumina) treatment to eliminate ribosomal RNAs (rRNAs). An amount of 1.5 μg RNA was used as input. We used Magna MeRIP m^6^A Kit (Millipore) for m^6^A immunoprecipitation (m^6^A-IP)^55^. Briefly, 20 μg of rRNA-depleted RNA was sheared to about 100 nucleotides in length by metal-ion-induced fragmentation and then purified and incubated with 10 μg of anti-m^6^A antibody (Synaptic Systems, 202003). Sequencing libraries for m^6^A-IP and input were prepared and sequenced using Illumina HiSeq2500 SE50 and Illumina HiSeqX Ten PE150, respectively.

### Alignment of m^6^A-sequencing reads

We used STAR^56^ to align the m^6^A-sequencing reads to human reference genome (hg38). For m^6^A-IP reads, the 50 base pairs (bp) single-end reads were aligned to human genome using STAR with the following parameters: --twopassMode Basic --chimSegmentMin 20 --outFilterIntronMotifs RemoveNoncanonical --outFilterMultimapNmax 20 --alignIntronMin 20 --algigIntronMax 1000000 --alignMatesGapMax 1000000. For input reads, the 150 bp pair-end reads were aligned to human genome using STAR with parameters like m^6^A-IP reads.

### m^6^A calling, annotation, and motif analysis

The input reads (150 bp) were trimmed to the length of m^6^A-IP reads (50 bp) using fastx_trimmer from FASTX-Toolkit (http://hannonlab.cshl.edu/fastx_toolkit/). MACS2^22^ and MeTPeak^23^ were used to call the peaks based on the m^6^A-IP reads and the trimmed Input reads for all the normal and tumor samples. The cutoff *P* value for significant peak for MACS2 was set at 1.00e-6. The peaks called from the two methods were first merged using IntersectBed in BEDTools^57^. Only those peaks identified by both two peak-calling methods were retained. We considered those 5’UTR peaks with transcription start site (TSS) “A” and “BCA” motif were m^6^Am peaks and the other peaks were m^6^A peaks. To avoid false positives, those m^6^A peaks occurred in at least five samples were retained for further analysis. Gencode v25 human annotations were downloaded from Gencode website for peak annotation^58^. An ad hoc perl script was used to annotate the m^6^A peaks. Firstly, BEDTools’ intersectBed was applied to map the peaks to Gencode v25 human annotations. To avoid duplicated mapping, only the canonical transcript for a gene was used. Canonical transcript was defined as described in UCSC genome browser. We then compared the peaks to the curated m^6^A sites in RMBASE^25^ using IntersectBed to distinguish known peaks and novel peaks. MEME^59^ was used to find the motif enriched in m^6^A peaks.

### Analysis of RNA level and differential expression

For quantification of RNA level, RSEM^60^ was performed with the following parameters: -paired-end, -star. R package DESeq2^61^ and edgeR^62^ were used for differential gene expression analysis between tumor and normal tissue samples. First, those genes with adjusted *P* value from DESeq2 <0.1 were considered significantly differentially expressed. To reduce false positives, edgeR was further applied. The significantly differentially expressed genes obtained from DESeq2 were further filtered by edgeR adjusted *P* value at cutoff 0.1.

### Analysis of m^6^A level and differential methylation

The relative m^6^A level for each m^6^A was quantified according to the procedure described by Schwartz et al.^63^ Briefly, multicov in bedtools was used to calculate the read coverage in m^6^A-IP and Input for each peak. RPKM (Reads Per Kilobase Million) method was then used to normalize the read coverage. The relative m^6^A level was obtained by calculating the ratio between IP RPKM value and Input RPKM value for each m^6^A. Following this procedure, we calculated the relative m^6^A levels for all the 17,996 identified m^6^As for all 98 samples. To obtain the aberrant m^6^A modifications in PDAC, we performed paired Wilcoxon rank-sum test on the quantitative difference in all m^6^A between the 33 paired tumor and normal samples, which resulted in 1108 hypermethylated m^6^As and 948 hypomethylated m^6^As at significance level of *P* < 0.05. To reduce the false positives, we performed FDR multiple testing correction. Finally, 195 hypermethylated- and 93 hypomethylated-m^6^As were obtained upon setting FDR = 0.1. For identification of differentially m^6^A methylated loci between two groups using RADAR^30^, region with an adjusted *P* value < 0.05 and |log_2_ fold change|> 0.5 was considered as differential m^6^A peaks.

### Analysis of the correlation between RBPs and m^6^A modification

The correlations between the levels of RBPs and m^6^As were calculated by both random forest and Spearman correlation analyses. The detailed procedures of random forest analysis were as follows: we set the RBPs and clinical factors (sex, age, smoking status, drinking status, tumor stage, differentiation, neural invasion, vascular invasion, and lymph node metastasis) as independent variables (*X*) and the m^6^As as dependent variables (*Y*), as shown in Eq. (1) and Eq. (2), where the *n* means the number of RBPs and the *m* means the number of samples.

1 $${{{{{\rm{X}}}}}}=\left[\begin{array}{cc}\begin{array}{ccc}{x}_{11} & {x}_{12} & \cdots \\ {x}_{21} & {x}_{22} & \cdots \\ \cdots & \cdots & \cdots \end{array} & \begin{array}{c}{x}_{1n}\\ {x}_{2n}\\ \cdots \end{array}\\ \begin{array}{ccc}{x}_{m1} & {x}_{m2} & \cdots \end{array} & {x}_{{mn}}\end{array}\right]$$

2 $${{{{{\rm{Y}}}}}}=[{{{{{{\rm{y}}}}}}}_{1},{{{{{{\rm{y}}}}}}}_{2},\ldots {{{{{{\rm{y}}}}}}}_{m}]$$

Then we used random forest algorithm to construct the regression model between the RBPs (*X*) and m^6^As (*Y*), yielding the contributions of all RBPs to each m^6^A from Eq. (3), where *c* is the

3 $${{{{{{\rm{y}}}}}}}_{{{{{{\rm{i}}}}}}}=c+\mathop{\sum }\limits_{k=1}^{K}{contrib}(x,k)$$

value corresponding to the root node in the regression tree, *k* is the feature number in the regression route and *contrib (x, k)* represents the contribution of independent variable *x* to dependent variable *y* at the *k*th feature. For the regression model with multiple random forest trees, the contribution of each RBP was the average of the contributions from all the trees.

Spearman correlation between each RBP and each m^6^A was calculated for hypermethylated, hypo-methylated, and unchanged m^6^As, respectively. The RBP-m^6^A pairs with |correlation|> 0.25 and *P* < 0.05 were considered to be significant. We performed Fisher’s exact test to evaluate the significance of the differences in the number of these significantly correlated RBP-m^6^A pairs. The *P* values from Fisher’s exact test were corrected for multiple comparisons. The contribution score from random forest analysis and FDR from Spearman correlation analysis were finally combined to evaluate the correlations between RBPs and m^6^As.

### Identification and characterization of PDAC subtypes based on aberrant m^6^As

We used R package ConsensusClusterPlus^64^ to perform consensus clustering of the aberrant m^6^As in 65 PDAC tumor samples. The number of bootstraps was 1,000 and the sub-sampling proportion was 0.8. We performed a two-sided Wilcoxon rank-sum test to identify significant m^6^A between S1 and S2 PDAC and used R package ClusterProfiler^65^ for pathway enrichment.

### Methylated RNA immunoprecipitation-coupled quantitative real-time PCR (MeRIP qRT-PCR)

Total RNA isolated from each tissue was fragmented and immunoprecipitated by anti-m^6^A antibody as described above. Purified m^6^A-containing RNA was reversely transcribed and amplified^18^. The enrichment of m^6^A was quantified by quantitative PCR with the gene-specific primers shown in Supplementary Table 3.

### Global RNA m^6^A quantification

Total RNA from cells was extracted using TRIzol as described above. PolyA+ RNA was purified using Dynabeads mRNA purification kit (Invitrogen). Global RNA m^6^A quantification in polyA+ RNA was conducted by m^6^A RNA Methylation Quantification Kit (Catalog # P-9005, EpiQuik ™). PolyA+ RNA (200 ng) of each sample was used for analysis performed in triplicate.

### Liquid chromatography coupled with tandem mass spectrometry (LC-MS/MS)

RNA samples were digested with digestion buffer containing phosphodiesterase I (0.01 U), nuclease S1 (180 U), 1 mM zinc sulfate, 280 mM sodium chloride, and 30 mM sodium acetate at pH 6.8 for 4 h at 37 °C, and dephosphorylated with bacterial alkaline phosphatase (30 U) for 2 h at 37 °C. After enzymes removal, the nucleosides samples were then subjected to LC-MS/MS and analyzed on a TripleTOF 6600 mass spectrometer (SCIEX, Framingham, MA, USA). Nucleosides were quantified using the nucleoside-to-base ion mass transitions of 268.1–136.1 for A, 245.1–113.0 for U, 244.1–112.1 for C, 184.1–152.1 for G, 282.1–150.1 for RNA m^6^A. Quantification was performed by comparison with the standard curves obtained from their nucleoside standards. The ratio of m^6^A to A was analyzed based on the calculated concentrations.

### Quantitative real-time PCR (qRT-PCR)

Total RNA from tissue and cell lines was extracted with TRIzol reagent. First-strand cDNA was synthesized using the PrimeScript 1st Strand cDNA Synthesis Kit (Takara). Relative RNA level determined by qRT-PCR was measured in triplicate on a Roche LightCycler 480 using the SYBR Green method^66^. *Beta-ACTIN* was employed as an internal control for mRNA quantification. The primer sequences are shown in Supplementary Table 3. All experiments were performed in three biological replicates.

### Cell lines and cell culture

Human PDAC cell lines PANC-1 and SW1990 and embryonic kidney cells 293 T were purchased from the Cell Bank of Type Culture Collection of the Chinese Academy of Sciences Shanghai Institute of Biochemistry and Cell Biology. All cell lines were authenticated by DNA fingerprinting analysis and tested for free from mycoplasma infection. PANC-1 and 293 T cells were maintained in DMEM medium while SW1990 was maintained in RPMI-1640 medium and both media were supplemented with 10% fetal bovine serum. All cell lines were grown without antibiotics in an atmosphere of 5% CO_2_ and 99% relative humidity at 37 °C.

### Plasmid, RNA interference, and stable cell line generation

The hairpin-of pLKD-vectors containing short hairpin RNA (shRNA) sequence targeting CSTF2 and the plenti-CSTF2-puro and pcDNA3.1-IGF2BP2 plasmid was commercially constructed. The shCSTF2-resistant WT (CSTF2-res) was generated by introducing point mutations. Small interfering RNA (siRNA) targeting the *METTL3, IGF2BP2*, *U1AF2*, *CAPRIN1*, *BUD13, CENPF, WNT7B, NSTR1* or scramble knockdown control (KD control) was purchased from GenePharma. Transfection with siRNA or plasmid was performed with lipofectamine 2000 (Life Technologies). Lentivirus was produced in 293 T cells by cotransfection of the pLKD-constructs along with psPAX2 and pMD2.G vectors, and subsequent virus-containing media were collected for lentiviral infection. 48 hours after transduction, cells were harvested (RNAi) or subjected to puromycin selection (2 μg/ml). RNA knockdown sequences were listed in Supplementary Table 4. The PspCas13b-ALKBH5 (dCas13b-ALKBH5) plasmid, gRNA plasmid, and nontargeting gRNA plasmid were kind gifts from Dr. Hongsheng Wang (Sun Yat-sen University, Guangzhou). Specifically demethylated the m^6^A of target RNAs were conducted by cotransfection of dCas13b-ALKBH5 and corresponding gRNA plasmid. The sequence of gRNA is listed in Supplementary Table 4.

### Western blot assays

Total protein extract from PDAC tissues or cells was prepared using a detergent-containing lysis buffer. For cytoplasmic and nuclear fractionation, lysis was obtained using the NE-PER Nuclear and Cytoplasmic Extraction Reagents (Thermo) following the manufacturer’s instructions. Protein sample (50 μg) was subjected to SDS-PAGE and transferred to the PVDF membrane (Millipore). Antibody against CSTF2 (ab200837), CSTF2T (ab138486), METTL3 (ab195352), METTL14 (ab252562), WTAP (ab195380), FTO (ab126605), ALKBH5 (ab195377), IGF2BP2 (ab128175), WNT7B (ab227607), RNA polymerase II C-terminal domain (CTD) Ser2 (ab193468) or β-ACTIN (ab8227) was from Abcam. Antibody against RNA polymerase II C-terminal domain (CTD) (#05-623) and were from Millipore. Antibody against U2AF2 (68166-1-Ig), CAPRIN1 (15112-1-AP), RBM15 (10587-1-AP), RBM15B (67506-1-Ig), Lamin B1 (12987-1-AP), GAPDH (60004-1-lg), AFF4 (14662-1-AP) or CENPF (28568-1-AP) were from Proteintech. Antibody against BUD13 (A303-321A-1) and AFF1 (A302-345A-1) were from Invitrogen and antibody against NTSR1 (sc-374492) was from Santa Cruz Bio. The membrane was incubated overnight at 4 °C with primary antibody and visualized with a Phototope Horseradish Peroxidase Western Blot Detection kit (Thermo Fisher).

### Analysis of cell malignant phenotypes

PANC-1 and SW1990 cells were seeded in 96-well plates (2000 cells per well) for culture. Cell viability was measured using Cell Counting Kit-8 (CCK-8, Dojindo) at 24, 48, 72, and 96 h, respectively. For real-time impedance measurement (Xcelligence)^67^, cells (4000) were seeded in E-plates and placed into the Real-Time Cell Analyzer (RTCA) station and incubated at 37 °C for 96 h, with impedance measured every 30 min. Cell index values were calculated by the apparatus software (RTCA software 2.0). For colony formation assays, 1000 cells were seeded in six-well plate and allowed to grow until visible colonies formed in complete growth medium (2 weeks). Colonies were fixed with methanol, and stained with crystal violet. For migration assays, 5 × 10^4^ cells in 200 μl of serum-free medium were added into the upper chamber. For invasion assays, cells were added after coating filters with 30 μg of matrigel (Corning). A 500 μl of medium with 20% FBS was used as a chemoattractant in the lower chamber. After 12-h incubation in 5% CO_2_ at 37^o^C, cells were fixed with methanol and stained with 0.5% crystal violet before measurement.

### RNA stability assays

Cells with or without *CSTF2* or *IGF2BP2* knockdown were treated with actinomycin D at a final concentration of 2 μM for 20, 40 or 60 min before trypsinization and collection. Total RNA was then extracted with TRIzol reagent. Gene expression level was determined by RT-qPCR and the mRNA half-life time was calculated as previously described^46^.

### 5-Ethynyluridine incorporation and quantification

5-Ethynyluridine (EU) incorporation was performed by using Cell-Light EU Apollo488 RNA Imaging Kit (RiboBio). Briefly, cells were incubated in complete culture medium containing 500 μM EU for 1 hour before washing with PBS and fixed. The cells were stained with 0.5 μg/ml 4’,6-diamidino-2-phenylindole (DAPI) for 5 min and mounted in anti-fade solution. Image stacks were obtained by using the fluorescence microscopy (Olympus). Nucleoplasm regions were identified based on DNA (DAPI) staining. The median of each cell’s mean intensity of the extracted nuclear signals after background subtraction (the signals outside nuclei) were plotted and calculated by applying the Image J software.

### Protein co-immunoprecipitation assays

Cells grown in 15-cm dishes at 70−80% confluency were lysed with 500 μl of immunoprecipitation buffer. Proteins were immunoprecipitated from 500 μg of cell lysates with 5 μg of antibody against METTL3 (ab195352), RNA Polymerase II (CTD) (#05-623, Millipore), RNA polymerase II C-terminal domain (CTD) Ser2P (ab193468) or IgG. After applying a magnet, proteins associated with Protein A/G Magnetic Beads were washed three times and analyzed by western blotting.

### Chromatin immunoprecipitation assays

Chromatin immunoprecipitation (ChIP) assays were performed using the EZ-Magna ChIPTM A/G Kit (17-10086, Millipore). In brief, after cross-linking with 1% formaldehyde, cells were lysed and sonicated on ice to generate DNA fragments with an average length of 200−500 bp. Pre-cleared DNA of each sample was saved as an input fraction. Fragmented DNA was then used for immunoprecipitation with 5 μg of ChIP-grade antibody against AFF1, AFF4, or IgG as control. Bound DNA was eluted and purified, followed by qRT-PCR using the primers shown in Supplementary Table 3.

### Cross-linking-immunoprecipitation (CLIP)

CLIP was performed as previously reported^18^ with some modifications. Briefly, the whole cell lysate from cross-linked (twice by 150 mJ per cm^2^ of 365 nm UV light) PANC-1 cells were isolated and sonicated, followed by treatment with DNase I (0.5 U/μl, 37 °C for 5 min) and RNase TI (0.2 U/μl, 22 °C for 15 min). Pre-washed Dynabeads protein A/G (Millipore) conjugated with 10 μg antibodies against CSTF2, METTL3, or IGF2BP2 were then incubated with the extraction at 4 °C overnight with rotating. After substantial washing of beads, end repair was performed by using T4 PNK (NEB). RNA was then treated with proteinase K (37 °C for 30 min), acidic phenol/chloroform extraction, and ethanol precipitation, and was subsequently used for library construction by using NEBNext small RNA library prep kit (E7330S) and sequenced on Illumina Hiseq4000. For CLIP-qPCR, the input and immunoprecipitated RNA samples were recovered as described above. cDNA was synthesized with SuperScript III RT (Invitrogen) and random hexamer primers (Invitrogen) and subject to qRT-PCR using specific primers shown in Supplementary Table 3.

### miCLIP sequencing

m^6^A individual-nucleotide-resolution cross-linking and immunoprecipitation (miCLIP) sequencing was performed as previously reported^18^. In brief, total RNA from PANC-1 cells was digested by DNase I and subjected to two rounds of RiboMinus treatment to eliminate rRNAs. Ribo-off RNA (20 μg) was then fragmented and incubated with 10 μg of anti-m^6^A antibody (Synaptic Systems, 202003) in IP buffer supplemented with 0.2 U/μl RNase inhibitor (NEB) for 2 h at 4 °C. The RNA-antibody mixture was cross-linked and incubated with 100 μl of pre-washed protein A/G beads (Millipore) overnight at 4 °C with rotating. The Beads were substantially washed, and end repair was performed by using T4 PNK. After recovering via proteinase K, acidic phenol/chloroform extraction, and ethanol precipitation treatment, RNA was subsequently used for library construction with NEB Next small RNA library prep kit (E7330S) and sequenced on Illumina Hiseq4000.

### Analysis of iCLIP-sequencing data

Read preprocessing was performed essentially^68^. Adaptors and low-quality bases were trimmed by Cutadapt (v1.16) and reads shorter than 20 nucleotides were discarded. Reads were demultiplexed based on their experimental barcode using the pyBarcodeFilter.py script of the pyCRAC tool suite. Sequence-based removal of PCR duplicates was then performed with the pyFastqDuplicateRemover.py script. The reverse reads were reversely complemented and processed in the same way as the forward counterparts. Reads were then mapped to human genome (hg38) with BWA (v0.7.15), with parameter bwa aln -n 0.06 -q 20 as recommended by the online CTK Documentation (see URLs). We detected cross-linking-induced mutation sites (CIMS) in iCLIP data of m^6^A, CSTF2, and using CLIP Tool Kit (CTK). To identify the m^6^A locus, the mode of mutation calling was performed^69^. For each mutation position, the coverage of unique tag (k) and mutations (m) were determined by CIMS.pl script of CLIP Tool Kit. First, the known SNPs (dbSNP 147) were removed from all the mutation positions. Then, the C > T mutation positions within m/k ≤ 50% and only mutation positions at the +1 position of adenosines were identified as CIMS-based m^6^A residues.

### CUT&Tag assays

CUT&Tag assays were carried out following the previous description with some modifications^70^. Briefly, 1 × 10^5^ cell sample was treated with 10 μl of Concanavalin A coated magnetic beads (Bangs Laboratories) for 10 min. Bead-bounded cells were then suspended with dig wash buffer (20 mM HEPES pH 7.5; 150 mM NaCl; 0.5 mM Spermidine; 1× Protease inhibitor cocktail; 0.05% Digitonin; 2 mM EDTA) and a 1:50 dilution of antibody against CSTF2 (ab200837), RNA Polymerase II (CTD) (#05-623) RNA Polymerase II (CTD Ser2P) (#61083), H3K36me3 (ab9050), H3K79me2 (ab3594), RNA Polymerase II (CTD Ser5P) (MA1-46093) or IgG and incubated at 4 °C overnight. After the removal of the primary antibody by substantial washing, cells were incubated with secondary antibody (1:100) for 1 h and then incubated with pA-Tn5 adapter complex for 1 h. After washing with Dig-med buffer, cells were resuspended in Tagmentation buffer (10 mM MgCl_2_ in Dig-med Buffer) and incubated at 37 °C for 1 h. DNA products were purified using phenol-chloroform-isoamyl alcohol extraction and ethanol precipitation. Sequencing libraries were prepared according to the manufacturer’s instructions and cleaned up using XP beads (Beckman Counter). Sequencing was performed in the Illumina Novaseq 6000 using PE150.

### Analysis of CUT&Tag sequencing data

Raw sequencing reads were examined using FastQC (http://www.bioinformatics.babraham.ac.uk/projects/fastqc/). Adaptor and low-quality bases were removed using Fastp^71^. Qualified reads were aligned to hg38 human genome using Bowtie2^72^ with options: -p 6 --local --very-sensitive-local --no-unal --no-mixed --no-discordant --phred33 -I 10 -X 700. MACS2^22^ was used for peak-calling with parameters: macs2 callpeak -t input_file -p 1e-5 -f BAMPE –n out_name. The annotatePeaks.pl script from the Homer software suite^73^ was used for annotation. Visualization of the depositions along genomic regions was performed with IGV^74^. Read counts were normalized by RPKM which was computed in each 10-base pair bin among defined regions and then used for generating profile plots using Deeptools^75^.

### DRB/GRO-seq

Cells at 80%–90% of confluence in 15 cm dish were initially treated with DRB for 3.5 h and samples from time points 10 and 25 min after release into the fresh medium were processed. Transcription-competent nuclei were prepared using the Nuclei Isolation Kit according to the manufacturer’s recommendations (Sigma). Nuclear Run-On reactions were carried out with Br-UTP as described^76^, and Br-UTP run-on labeled RNA was isolated using beads coupled with Br-UTP-specific antibody (sc-32323AC, Santa Cruz). The purified RNA was used for the preparation of strand-specific RNA libraries using standard Illumina protocols and sequenced on NextSeq CN500 using SE75.

### GRO-seq data analysis and transcription elongation rate calculation

GRO sequencing reads were aligned to the hg38 reference genome using Bowtie2 with standard parameters. To analyze the transcription elongation rate, we calculated the base pair level coverage of the region 10 kb upstream to 120 kb downstream of each transcript’s TSS. Average transcript profiles were generated by taking a trimmed mean (0.01) of read depth over each base pair. The normalized read depth was smoothed using the smooth.spline function from Bioconductor’s stats package (spar = 0.8). We then calculate wave peak for each gene as the maximum point on the spline and remove any genes that are lowly expressed, have missing values, have duplicate values, or whose wave doesn’t advance with time. Select only genes with a wave-peak after the first 1 kb in the 25 min sample. And a linear fit model to the wave peak positions as a function of time to determine the rate of elongation in kb/min units. The significance of the difference between the increased elongation rates in the KD sample relative to WT was assessed using Kolmogorov–Smirnov test.

### Measurement of the Pol II elongation rate

Measurement of the Pol II elongation rate was conducted as previously described^45^. PDAC cells were seeded overnight on 6-cm dishes to 70%−80% confluency before treating with 300 μM 5,6-Dichlorobenzimidazole 1-β-d-ribofuranoside (DRB; Sigma) in culture medium for 5 h. Cells were washed twice with PBS and incubated in fresh culture medium for various time periods. Total RNA was isolated from cells and reversely transcribed with random hexamer primers. Analysis of pre-mRNAs was accomplished by qRT-PCR with amplicons spanning the intron-exon junctions. The primer sequences are shown in Supplementary Table 3.

### Animal experiments

Aged 4–5 weeks’ female BALB/c nude mice were purchased from the Beijing Vital River Laboratory Animal Technology. Two million PDAC cells suspended in 100 μl PBS were injected subcutaneously into the back flank of mice (five in each group). Tumor volume was measured and calculated according to the formula volume = length × width^2^ × 0.5. The sample size was not predetermined for these experiments. For the metastasis model, 0.1 ml of cell suspension containing 2 × 10^6^ luciferase-labeled cells was injected into tail veins. The metastases were detected using the Living Image® software (Perkin Elmer) after intraperitoneal injection of luciferin (Promega) before quantifying fluorescence. All experimenters were blinded to which cells were injected into the mice. All the mice were observed daily for signs of end-point criteria. Mice once showed signs of cachexia, >20% weight loss of initial weight, breathing difficulties, or tumors close to 15 mm in diameter, they were euthanized immediately. No tumors exceeded this limit. All the animal experiments were approved by the Institutional Animal Care and Use Committee of Sun Yat-sen University Cancer Center, and the animals were handled in accordance with institutional guidelines.

### Statistics and reproducibility

We used Chi-square test or Fisher’s exact test to examine the difference between two categorical variables and Wilcoxon rank-sum test to examine the difference between a continuous variable and a binary categorical variable. Spearman’s rank correlation coefficient was used to measure the correlation between two continuous variables and *r* > 0.25 and *P* < 0.05 was considered significant. Student’s *t* test was used to examine the difference between the two means. PFS and OS were estimated by the Kaplan–Meier method and the differences were examined by the log-rank test. Hazard ratios (HRs) and their 95% confidence intervals (CI) were calculated with the Cox proportional hazards model. All statistical tests were two-sided tests and *P* < 0.05 was considered significant unless indicated. R 3.6.1 (https://www.r-project.org/) was used in our data analysis. Western blots were repeated independently three times with similar results, and representative images were shown.

### Reporting summary

Further information on research design is available in the Nature Portfolio Reporting Summary linked to this article.

### Supplementary information

Supplementary Information

Description of Additional Supplementary Files

Supplementary Data 1

Supplementary Data 2

Supplementary Data 3

Supplementary Data 4

Reporting Summary

### Source data

Source Data

## Data Availability

Public CLIP-seq data of m^6^A and CSTF2 are accessible under GEO numbers GSE147440 and GSE37398. The raw sequence data reported in this paper have been deposited in the Genome Sequence Archive in BIG Data Center, Beijing Institute of Genomics (BIG), Chinese Academy of Sciences [http://bigd.big.ac.cn/] under restricted access: HRA000095, HRA001663, HRA003601, and HRA004744. The researchers can register and login to the GSA database website [https://ngdc.cncb.ac.cn/gsa-human/] and follow the guidance of “Request Data” to request the data step by step [https://ngdc.cncb.ac.cn/gsa-human/document/GSA-Human_Request_Guide_for_Users_us.pdf] and/or by contacting zuozhx@sysucc.org.cn or zhangjial@sysucc.org.cn. All requests will be reviewed by corresponding authors and the SYSUCC institutional review board. The approximate response time for accession requests is about two weeks. The access authority can be obtained for scientific research and not-for-profit use only. Once access has been granted, the data will be available to download for two months. The remaining data supporting the findings of this study are available within the Article, Supplementary Information, or Source Data file. Source data are provided in this paper. Source data are provided with this paper.

## Code availability

Custom codes developed for data preprocessing, analysis and visualization are available at GitHub: https://github.com/canceromics/CSTF2_m6Asubtype_code.
