# Supplementary material for: CSTF2 mediated mRNA N6-methyladenosine modification drives pancreatic ductal adenocarcinoma m6A subtypes
Source: Nat Commun. 2023 Oct 10;14:6334. doi: 10.1038/s41467-023-41861-y (PMC10564946; doi:10.1038/s41467-023-41861-y)
Supplement: Supplementary file 1 — Supplementary Information [file 41467_2023_41861_MOESM1_ESM.pdf]

Supplementary Material for
CSTF2 mediated mRNA N^6-methyladenosine modification drives pancreatic ductal adenocarcinoma m^6A subtypes

This PDF includes:

Supplementary Figures

Supplementary Fig. 1. The distribution of m^6As in PDAC and adjacent normal samples and annotation of the dysregulated m^6As.
Supplementary Fig. 2. Characters of two PDAC subtypes and integrated analysis with reported PDAC subtype.
Supplementary Fig. 3. High expression of CSTF2 contributes to hyper-methylated m^6A in PDAC.
Supplementary Fig. 4. Knockdown of CSTF2 inhibits proliferation and metastasis of PDAC cells.
Supplementary Fig. 5. CSTF2 promotes mRNA-m^6A modifications co-transcriptionally.
Supplementary Fig. 6. CSTF2 promotes mRNA-m^6A via slowing elongation.
Supplementary Fig. 7. CSTF2-induced moderative elongation facilitates METTL3-catalyzed m^6A deposition.
Supplementary Fig. 8. Hyper m^6A methylation enhances mRNA stability.
Supplementary Fig. 9. Effects of m^6A level alteration by using the dCas13 based m^6A editing system.

Supplementary Tables

Supplementary Table 1. Baseline demographic and clinical characteristics of PDAC patients in this study.
Supplementary Table 2. Validation results of selected differentially methylated m^6As between PDAC and normal tissues by MeRIP qRT-PCR method.
Supplementary Table 3. The primers sequences used in this study.
Supplementary Table 4. The RNA knockdown and gRNA sequences used in this study.

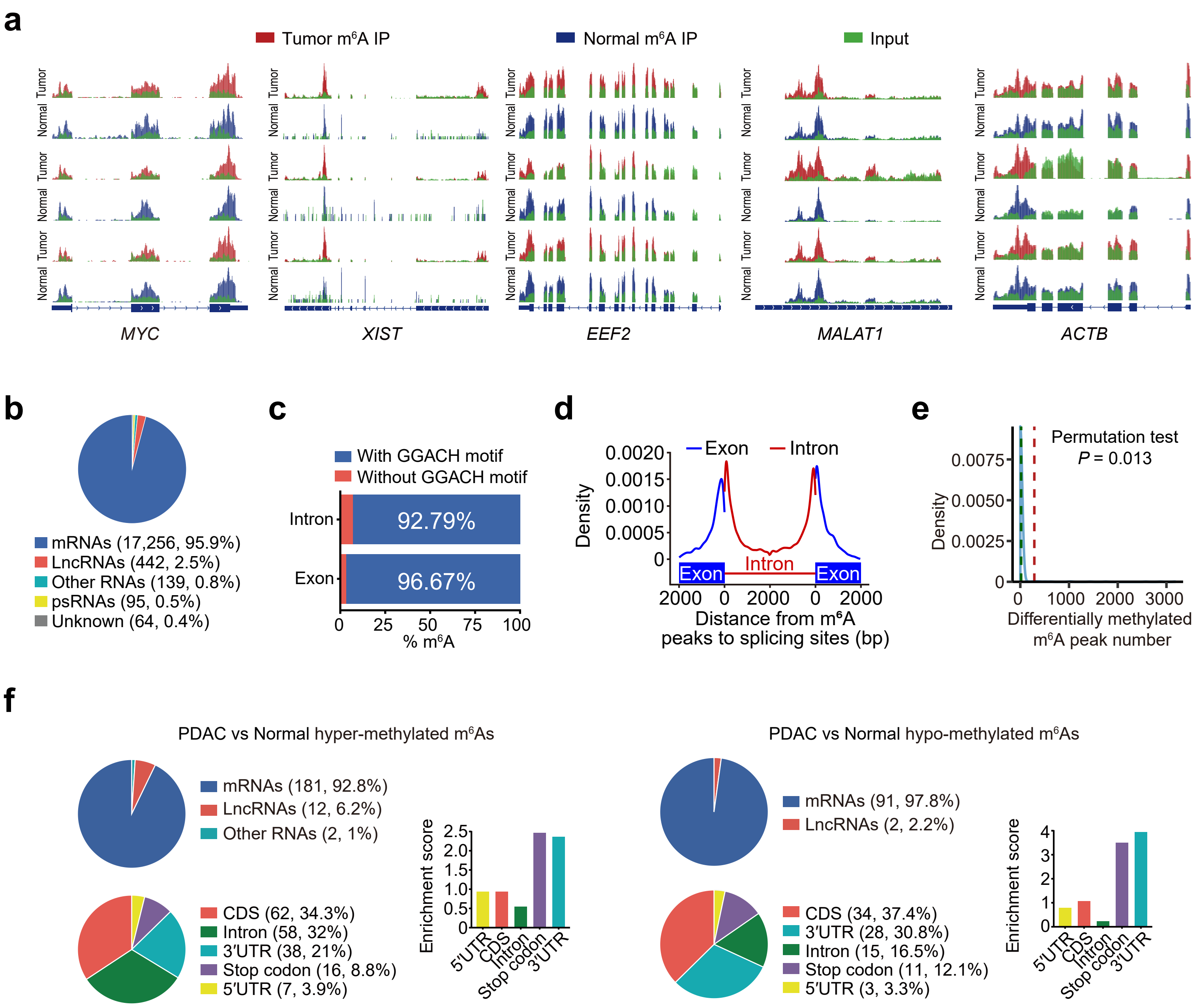

Supplementary Fig. 1. The distribution of m⁶As in PDAC and adjacent normal samples and annotation of the dysregulated m⁶As.

a The abundance of m⁶A in *MYC*, *XIST*, *EEF2*, *MALAT1* and *ACTB* mRNAs that are well-known to have m⁶A modifications. **b** Percentage of annotated mRNAs, long non-coding RNAs (lncRNAs), pseudogene RNAs (psRNAs), other type RNAs and unknown. **c** GGACH motif distribution in exon-derived or intron-derived m⁶As. **d** Distance between m⁶A peak and splicing site. **e** Density plot of the distribution of differentially methylated m⁶As number resulted from 1,000 permutation test. The red dash line represents observed number while the green dash line represents the average number of 1,000 time permutation. *P* value was calculated from permutation test. **f** The proportion of hyper-methylated m⁶As (*left panel*) and hypo-methylated m⁶As (*right panel*) in mRNA, lncRNA and other RNAs or in 5'UTR, CDS, 3'UTR, stop-codon of mRNAs and intron region.

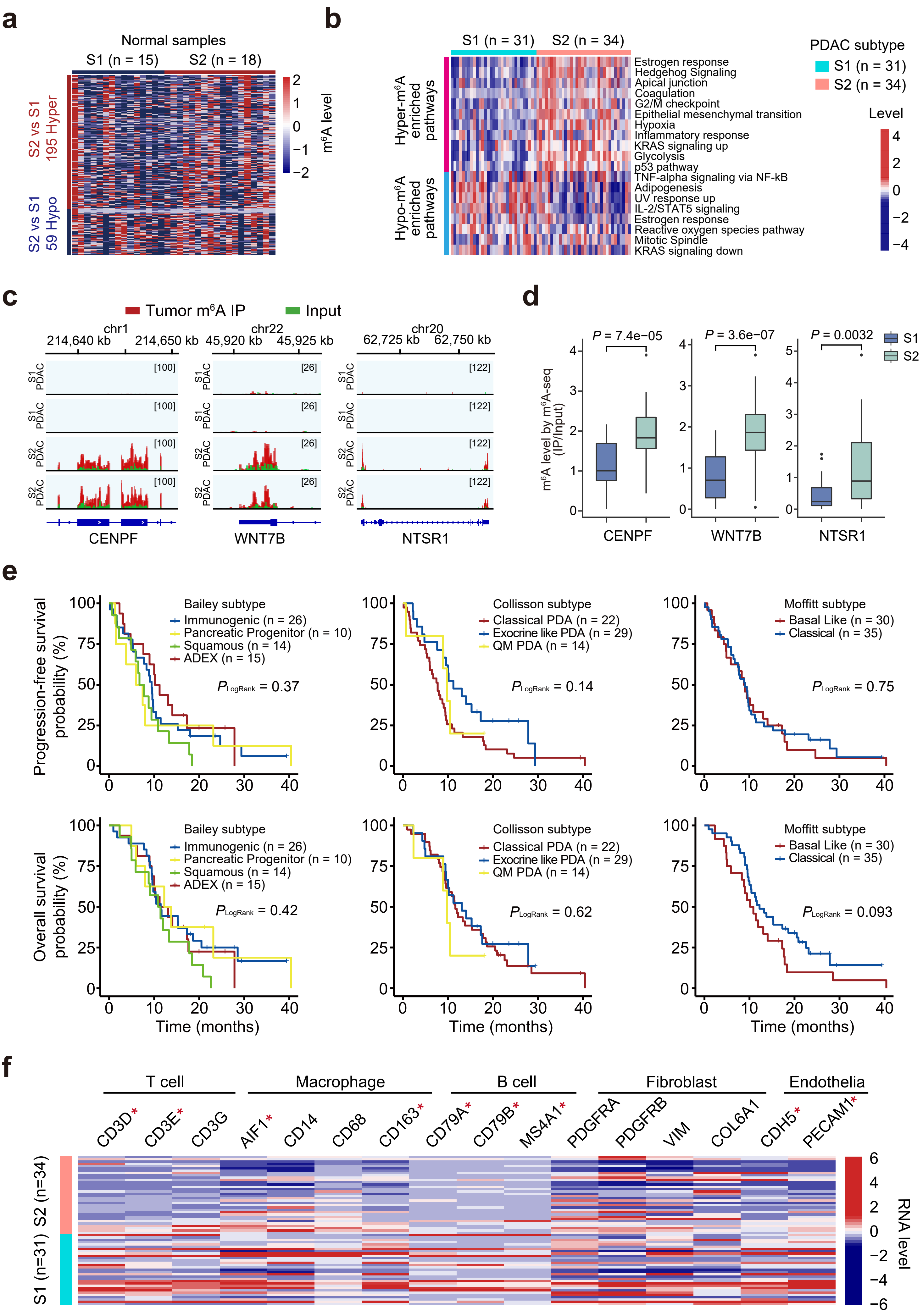
a
Normal samples
S1 (n = 15)
S2 (n = 18)
S2 vs S1 195 Hyper
S2 vs S1 59 Hypo
m6A level
b
S1 (n = 31)
S2 (n = 34)
Hyper-m6A enriched pathways
Hypo-m6A enriched pathways
Estrogen response
Hedgehog Signaling
Apical junction
Coagulation
G2/M checkpoint
Epithelial mesenchymal transition
Hypoxia
Inflammatory response
KRAS signaling up
Glycolysis
p53 pathway
TNF-alpha signaling via NF-kB
Adipogenesis
UV response up
IL-2/STAT5 signaling
Estrogen response
Reactive oxygen species pathway
Mitotic Spindle
KRAS signaling down
PDAC subtype
S1 (n = 31)
S2 (n = 34)
Level
c
Tumor m6A IP
Input
chr1
214,640 kb
214,650 kb
chr22
45,920 kb
45,925 kb
chr20
62,725 kb
62,750 kb
S1 PDAC
S2 PDAC
CENPF
WNT7B
NTSR1
d
P = 7.4e−05
P = 3.6e−07
P = 0.0032
m6A level by m6A-seq (IP/Input)
S1
S2
CENPF
WNT7B
NTSR1
e
Progression-free survival probability (%)
Bailey subtype
Immunogenic (n = 26)
Pancreatic Progenitor (n = 10)
Squamous (n = 14)
ADEX (n = 15)
PLogRank = 0.37
Collisson subtype
Classical PDA (n = 22)
Exocrine like PDA (n = 29)
QM PDA (n = 14)
PLogRank = 0.14
Moffitt subtype
Basal Like (n = 30)
Classical (n = 35)
PLogRank = 0.75
Overall survival probability (%)
PLogRank = 0.42
PLogRank = 0.62
PLogRank = 0.093
Time (months)
f
T cell
Macrophage
B cell
Fibroblast
Endothelia
CD3D*
CD3E*
CD3G
AIF1*
CD14
CD68
CD163*
CD79A*
CD79B*
MS4A1*
PDGFRA
PDGFRB
VIM
COL6A1
CDH5*
PECAM1*
S2 (n=34)
S1 (n=31)
RNA level

Supplementary Fig. 2. Characters of two PDAC subtypes and integrated analysis with reported PDAC subtype.

a Heatmap showing the expression level of different m^6A (S2 vs S1 PDAC, FDR < 0.1 of Wilcoxon rank-sum test) in normal tissues. **b** Heatmap showing the differential m^6A level of gene sets in indicated pathways in S1 and S2 PDAC. **c** The abundance of m^6A in *CENPF*, *WNT7B*, and *NTSR1* mRNAs that are detected by m^6A-seq in S1 and S2 PDAC. Number represents the range of the m^6A signals. **d** The abundance of m^6A in *CENPF*, *WNT7B*, and *NTSR1* mRNAs that are qualified by m^6A-seq in S1 PDAC (n = 31) and S2 PDAC (n = 34). Boxplots indicate median (middle line), 25th, 75th percentile (box) and 5th and 95th percentile (whiskers). *P* values were from Wilcoxon rank-sum test. **e** Kaplan-Meier estimates of progression-free survival (*upper panel*) and overall survival (*lower panel*) in patients with PDAC by previously reported transcriptional subtypes in our patient set. **f** Heatmap showing the differences in mRNA levels of indicated microenvironmental cell markers in S1 and S2 PDAC subtypes. *, $P < 0.05$ of Wilcoxon rank-sum test.

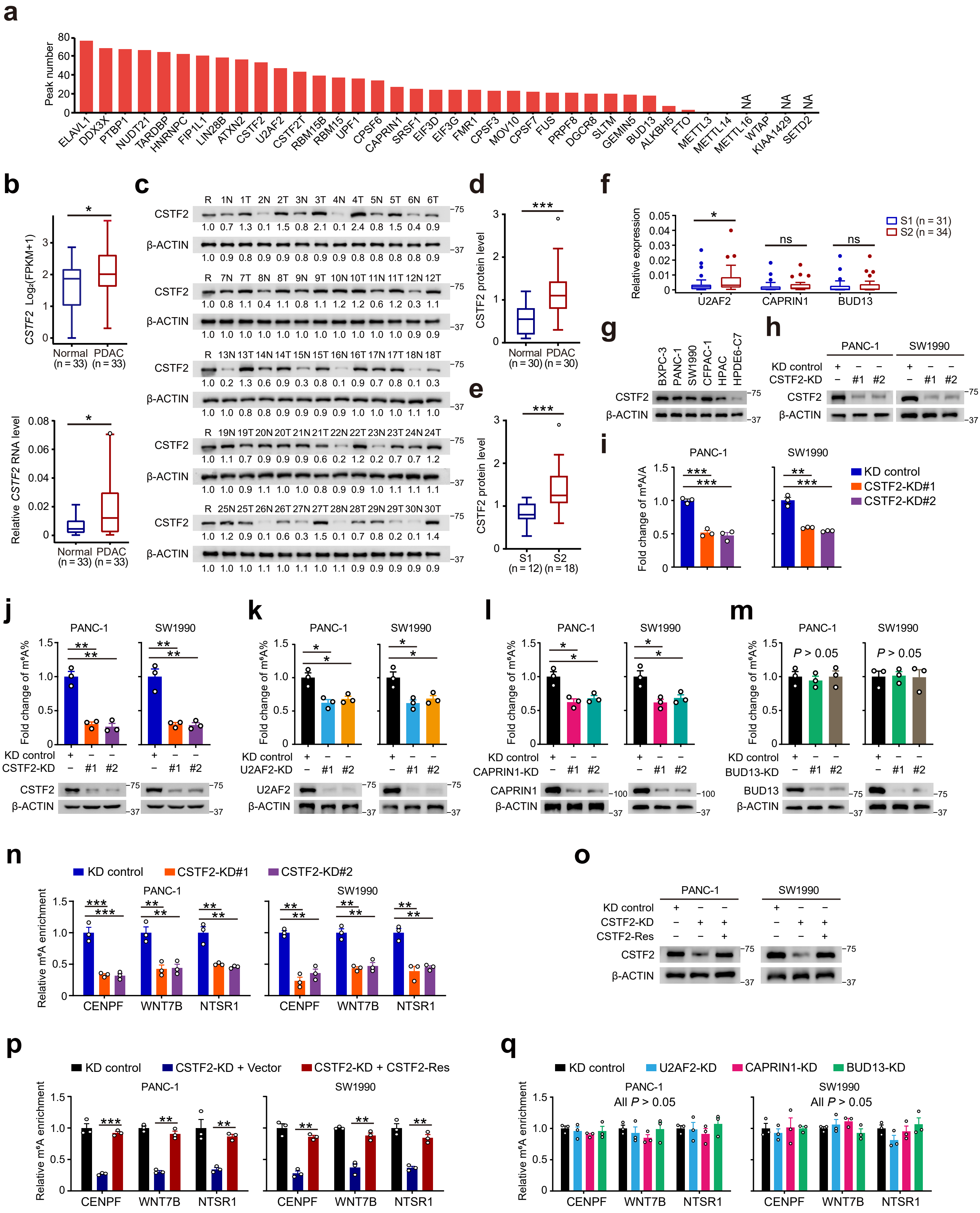

a
Peak number
ELAVL1 DDX3X PTBP1 NUDT21 TARDBP HNRNPC FIP1L1 LIN28B ATXN2 CSTF2 U2AF2 CSTF2T RBM15B RBM15 UPF1 CPSF6 CAPRIN1 SRSF1 EIF3D EIF3G FMR1 CPSF3 MOV10 CPSF7 FUS PRPF8 DGCR8 SLTM GEMIN5 BUD13 ALKBH5 FTO METTL3 METTL14 METTL16 WTAP KIAA1429 SETD2
NA
b
CSTF2 Log2(FPKM+1)
Normal PDAC
(n = 33) (n = 33)
Relative CSTF2 RNA level
c
CSTF2
β-ACTIN
R 1N 1T 2N 2T 3N 3T 4N 4T 5N 5T 6N 6T
1.0 0.7 1.3 0.1 1.5 0.8 2.1 0.1 2.4 0.8 1.5 0.4 0.9
1.0 0.9 0.8 0.9 0.9 0.8 0.9 0.9 0.8 0.8 0.8 0.8 0.9
R 7N 7T 8N 8T 9N 9T 10N 10T 11N 11T 12N 12T
1.0 0.8 1.1 0.4 1.1 0.8 1.1 1.2 1.2 0.6 1.2 0.3 1.1
1.0 1.0 1.0 1.0 1.0 1.0 1.0 1.0 1.0 1.0 1.0 0.9 0.9
R 13N 13T 14N 14T 15N 15T 16N 16T 17N 17T 18N 18T
1.0 0.2 1.3 0.6 0.9 0.3 0.8 0.1 0.9 0.7 0.8 0.1 0.3
1.0 1.0 0.8 0.8 0.9 0.9 0.9 0.9 1.0 1.0 1.0 1.1 1.0
R 19N 19T 20N 20T 21N 21T 22N 22T 23N 23T 24N 24T
1.0 1.1 0.7 0.9 0.9 0.9 0.6 0.2 1.2 0.2 0.7 0.7 1.2
1.0 1.0 0.9 1.1 1.0 1.0 0.8 0.9 0.9 1.0 1.1 1.1 1.1
R 25N 25T 26N 26T 27N 27T 28N 28T 29N 29T 30N 30T
1.0 1.2 0.9 0.1 0.6 0.3 1.5 0.1 0.7 0.8 0.2 0.1 1.4
1.0 1.0 1.0 1.1 1.0 1.1 1.0 1.1 0.9 0.9 0.9 0.9 0.9
d
CSTF2 protein level
Normal PDAC
(n = 30) (n = 30)
e
CSTF2 protein level
S1 S2
(n = 12) (n = 18)
f
Relative expression
S1 (n = 31)
S2 (n = 34)
U2AF2 CAPRIN1 BUD13
ns
g
BXPC-3 PANC-1 SW1990 CFPAC-1 HPAC HPDE6-C7
CSTF2
β-ACTIN
h
PANC-1 SW1990
KD control
CSTF2-KD
#1 #2
i
PANC-1 SW1990
Fold change of m6A/A
KD control
CSTF2-KD#1
CSTF2-KD#2
j
Fold change of m6A%
KD control
CSTF2-KD
k
U2AF2-KD
U2AF2
l
CAPRIN1-KD
CAPRIN1
m
P > 0.05
BUD13-KD
BUD13
n
Relative m6A enrichment
CENPF WNT7B NTSR1
o
CSTF2-Res
p
CSTF2-KD + Vector
CSTF2-KD + CSTF2-Res
q
U2AF2-KD CAPRIN1-KD BUD13-KD
All P > 0.05

Supplementary Fig. 3. High expression of CSTF2 contributes to hyper-methylated m^6A in PDAC.

a The identified hyper-methylated m^6A peak number for different RNA binding proteins (RBPs). The peak number for each RBP was calculated based on whether the genomic regions of the peak and RNA binding site of the specific RBP have any overlap (1 bp). **b** *CSTF2* expression levels of paired PDAC tumor and normal samples in our data determined by RNA-sequencing (*upper panel*) or qRT-PCR (*lower panel*). *, $P < 0.05$ of Wilcoxon rank-sum test. **c** Western blot analysis of CSTF2 levels in 30 paired PDAC tumor and normal samples. R was the same positive reference sample used for loading control on each gel. Each protein band was quantified by gray density and the value for each band is relative to density of both β-ACTIN and the corresponding R band. **d**, **e** *CSTF2* expression levels in PDAC tumor and normal samples (**d**) and S1 and S2 PDAC subtypes (**e**) determined by western blot data from (**c**). ***, $P < 0.001$ of Wilcoxon rank-sum test. **f** The expression levels of indicated transcript in S1 and S2 PDAC in our data determined by qRT-PCR. *BUD13* serves as negative control. *, $P < 0.05$; ns, not significant of Wilcoxon rank-sum test. **g** Western blot analysis of CSTF2 levels in PDAC cells and normal pancreas cell line. **h** Knockdown efficiency was verified by western-blot. **i** The effect of *CSTF2* expression change on m^6A level in PANC-1 and SW1990 cells determined by m^6A-LC-MS. **, $P < 0.01$; ***, $P < 0.001$ of Student's *t*-test compared with KD control. **j−m** The effect of CSTF2 (**j**), U2AF2 (**k**), CAPRIN1 (**l**), BUD13 (**m**) knockdown on m^6A level in PANC-1 and SW1990 cells determined by m^6A-ELISA. BUD13 serves as negative control. **n** The abundance of m^6A in *CENPF*, *WNT7B*, and *NTSR1* mRNAs that are detected by MeRIP-qPCR in PANC-1 with or without *CSTF2* KD. **o** Western blot analysis of CSTF2 levels in PDAC cells upon *CSTF2* KD with or without rescued with shRNA-resistant CSTF2. **p** The abundance of m^6A in *CENPF*, *WNT7B*, and *NTSR1* mRNAs that are detected by MeRIP-qPCR in PDAC cells upon *CSTF2* KD with or without rescued with shRNA-resistant CSTF2. **q** The abundance of m^6A in *CENPF*, *WNT7B*, and *NTSR1* mRNAs that are detected by MeRIP-qPCR in PDAC cell line upon *U2AF2* KD, *CAPRIN1* KD and *BUD13* KD. Boxplots in **b**, **d−f** indicate median (middle line), 25th, 75th percentile (box) and 5th and 95th percentile (whiskers). Data are the mean ± S.E.M. of three independent experiments in **i−n**, **p** and **q**. *, $P < 0.05$ and **, $P < 0.01$ of Student's *t*-test compared with each control.

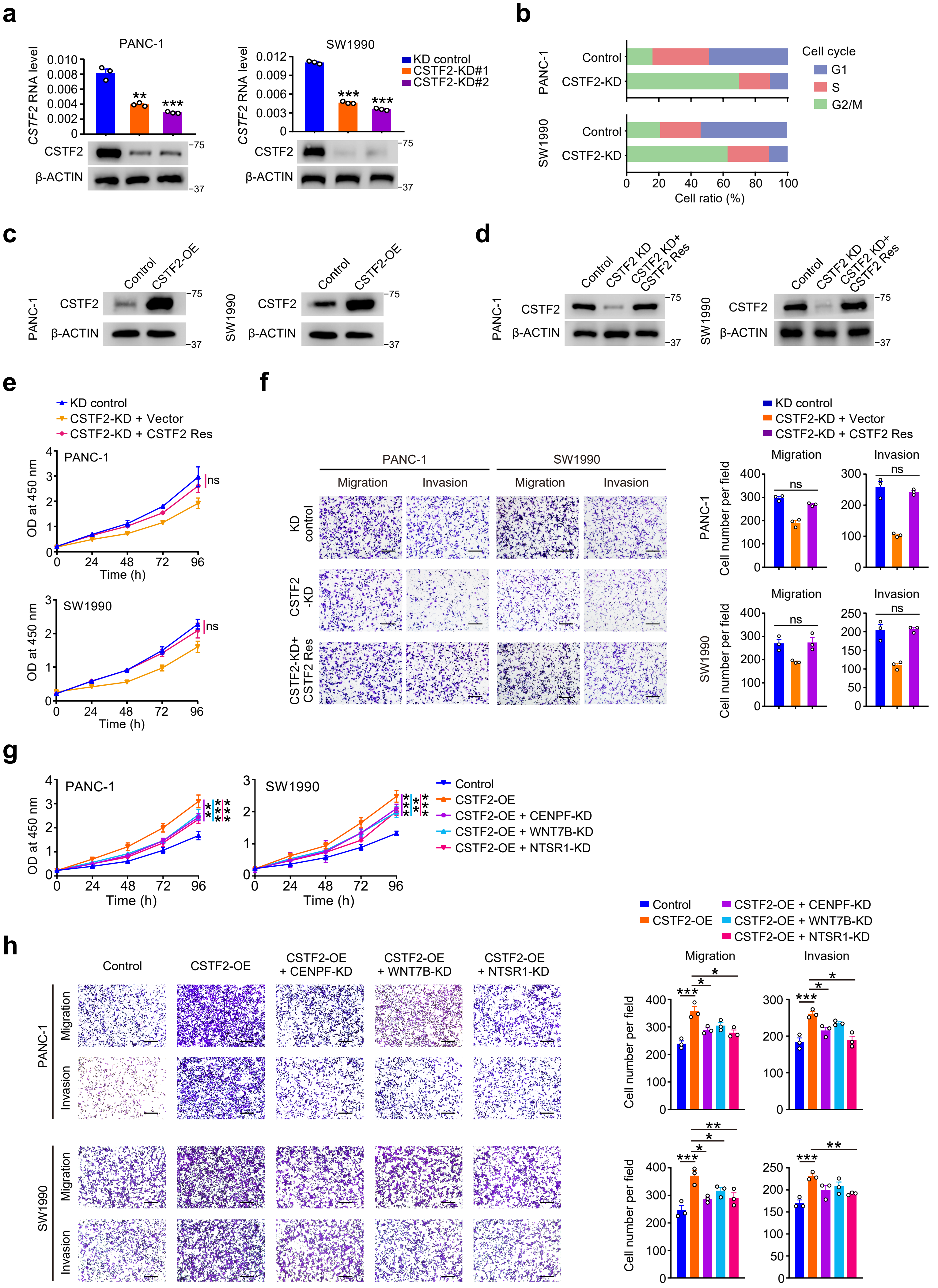
a
PANC-1
SW1990
CSTF2 RNA level
KD control
CSTF2-KD#1
CSTF2-KD#2
CSTF2
β-ACTIN
b
Control
CSTF2-KD
PANC-1
SW1990
Cell cycle
G1
S
G2/M
Cell ratio (%)
c
Control
CSTF2-OE
d
CSTF2 KD
CSTF2 KD+ CSTF2 Res
e
KD control
CSTF2-KD + Vector
CSTF2-KD + CSTF2 Res
OD at 450 nm
Time (h)
ns
f
Migration
Invasion
KD control
CSTF2 -KD
CSTF2-KD+ CSTF2 Res
Cell number per field
g
Control
CSTF2-OE
CSTF2-OE + CENPF-KD
CSTF2-OE + WNT7B-KD
CSTF2-OE + NTSR1-KD
h
CSTF2-OE + CENPF-KD
CSTF2-OE + WNT7B-KD
CSTF2-OE + NTSR1-KD

Supplementary Fig. 4. Knockdown of CSTF2 inhibits proliferation and metastasis of PDAC cells.

a *CSTF2* expression levels in PANC-1 and SW1990 cells with or without *CSTF2* KD. **b** Cell cycle profiling shows the effect of *CSTF2* KD on cell cycle distribution. **c** *CSTF2* expression levels in PANC-1 and SW1990 cells with or without forced *CSTF2* expression. **d** Western blot analysis of CSTF2 in PDAC cells upon *CSTF2* KD with or without rescued with shRNA-resistant CSTF2. **e, f** Inhibition of proliferation (**e**), migration and invasion (**f**) upon *CSTF2* KD can be rescued by shRNA-resistant CSTF2. **g, h** Malignant phenotypes promoted by forced CSTF2 expression can be partially rescued by knockdown of specific CSTF2-m^6A target (*CENPF, WNT7B, NTSR1*) respectively. Data are the mean ± S.E.M. of three independent experiments. Scale bars, 200 μm.*, *P* < 0.05; **, *P* < 0.01 and ***, *P* < 0.001 of Student's *t*-tests compared with each control.

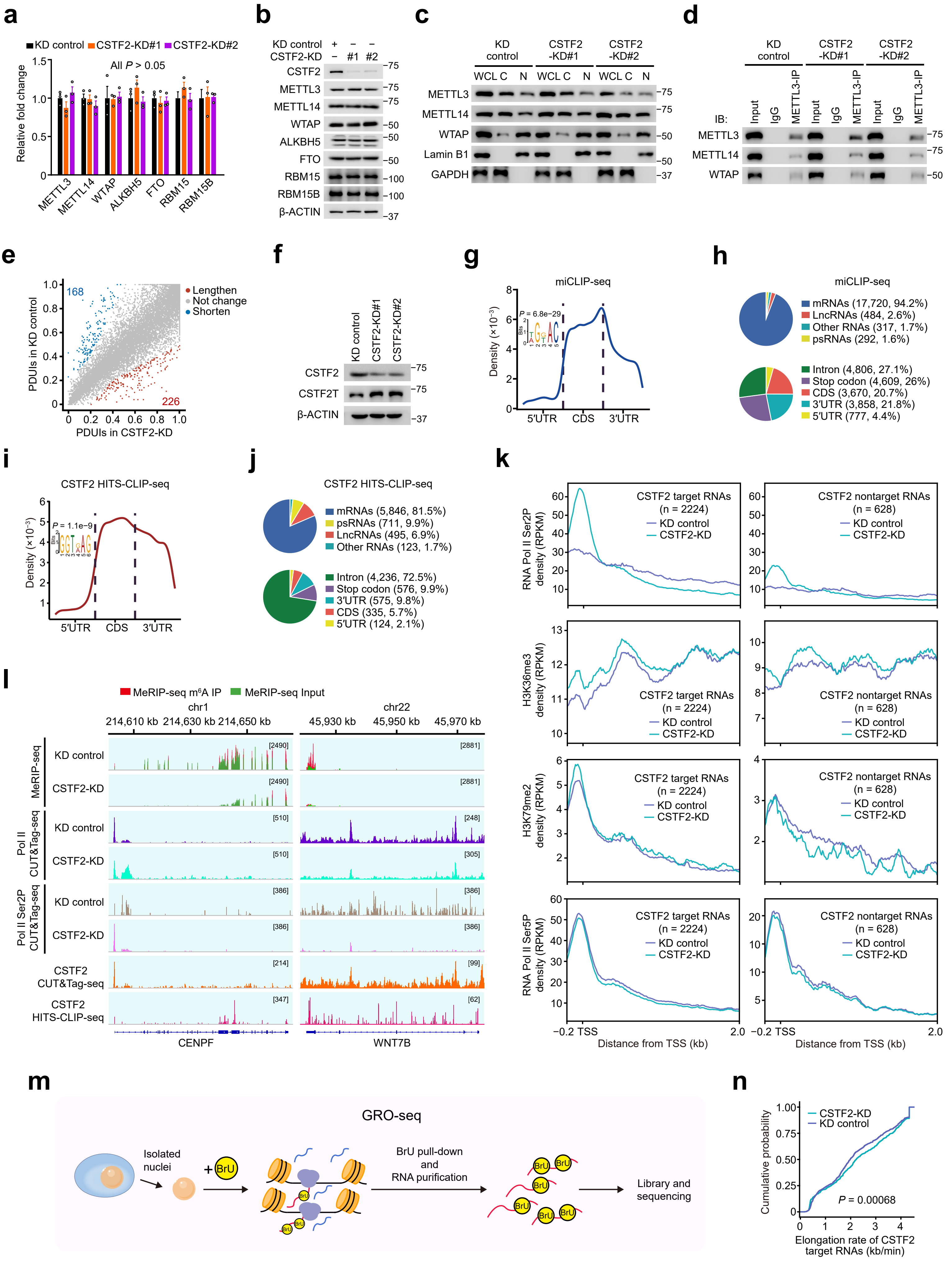
a
KD control CSTF2-KD#1 CSTF2-KD#2
All P > 0.05
Relative fold change
METTL3 METTL14 WTAP ALKBH5 FTO RBM15 RBM15B
b
KD control + − −
CSTF2-KD − #1 #2
CSTF2 METTL3 METTL14 WTAP ALKBH5 FTO RBM15 RBM15B β-ACTIN
c
KD control CSTF2 -KD#1 CSTF2 -KD#2
WCL C N
METTL3 METTL14 WTAP Lamin B1 GAPDH
d
KD control CSTF2 -KD#1 CSTF2 -KD#2
Input IgG METTL3-IP
IB:
METTL3 METTL14 WTAP
e
168
226
Lengthen
Not change
Shorten
PDUIs in KD control
PDUIs in CSTF2-KD
f
KD control CSTF2-KD#1 CSTF2-KD#2
CSTF2 CSTF2T β-ACTIN
g
miCLIP-seq
P = 6.8e−29
Density (×10⁻³)
5′UTR CDS 3′UTR
h
miCLIP-seq
mRNAs (17,720, 94.2%)
LncRNAs (484, 2.6%)
Other RNAs (317, 1.7%)
psRNAs (292, 1.6%)
Intron (4,806, 27.1%)
Stop codon (4,609, 26%)
CDS (3,670, 20.7%)
3′UTR (3,858, 21.8%)
5′UTR (777, 4.4%)
i
CSTF2 HITS-CLIP-seq
P = 1.1e−9
Density (×10⁻³)
5′UTR CDS 3′UTR
j
CSTF2 HITS-CLIP-seq
mRNAs (5,846, 81.5%)
psRNAs (711, 9.9%)
LncRNAs (495, 6.9%)
Other RNAs (123, 1.7%)
Intron (4,236, 72.5%)
Stop codon (576, 9.9%)
3′UTR (575, 9.8%)
CDS (335, 5.7%)
5′UTR (124, 2.1%)
k
CSTF2 target RNAs (n = 2224)
CSTF2 nontarget RNAs (n = 628)
KD control
CSTF2-KD
RNA Pol II Ser2P density (RPKM)
H3K36me3 density (RPKM)
H3K79me2 density (RPKM)
RNA Pol II Ser5P density (RPKM)
−0.2 TSS 2.0
Distance from TSS (kb)
l
MeRIP-seq m⁶A IP MeRIP-seq Input
chr1 chr22
214,610 kb 214,630 kb 214,650 kb
45,930 kb 45,950 kb 45,970 kb
MeRIP-seq
Pol II CUT&Tag-seq
Pol II Ser2P CUT&Tag-seq
KD control
CSTF2-KD
CSTF2 CUT&Tag-seq
CSTF2 HITS-CLIP-seq
CENPF WNT7B
m
GRO-seq
Isolated nuclei
BrU
BrU pull-down and RNA purification
Library and sequencing
n
CSTF2-KD
KD control
Cumulative probability
P = 0.00068
Elongation rate of CSTF2 target RNAs (kb/min)

Supplementary Fig. 5. CSTF2 promotes mRNA-m^{6}A modifications co-transcriptionally.

a and **b** Effects of *CSTF2* knockdown in PANC-1 cells on mRNA and protein levels of known writers (METTL3, METTL14, WTAP) and erasers (FTO, ALKBH5, RBM15, RBM15B) detected by qRT-PCR (**a**) or western blot (**b**). Values are mean ± S.E.M. in n = 3 independent experiments in **a**. **c** The subcellular distribution of methyltransferase complex upon *CSTF2* KD. **d** The intact methyltransferase complex was not affected upon *CSTF2* KD. **e** Global APA profiling in PANC-1 upon *CSTF2* KD. **f** Western blot analysis of CSTF2T in PANC-1 upon *CSTF2* KD. **g** Metagene analysis of the m^{6}A sites identified in miCLIP sequencing in PANC-1 cells. **h** The proportions of m^{6}A sites in mRNAs, lncRNAs, pseudogene RNAs and other type RNAs (*upper panel*) and in the 5'UTR, CDS, 3'UTR and stop-codon of mRNAs and intron (*lower panel*). **i** Metagene analysis of CSTF2-binding sites identified in CSTF2 HITS-CLIP sequencing in PANC-1 cells. **j** The proportion of CSTF2-binding sites in mRNAs, lncRNAs, pseudogene RNAs and other type RNAs (*upper panel*) and in the 5'UTR, CDS, 3'UTR and stop-codon of mRNAs and intron (*lower panel*). **k** Comparison of the RNA Pol II-Ser2P, H3K36me3, H3K79me2 and RNA Pol II-Ser5P density along the mRNA (*left*: CSTF2 target RNAs; *right*: CSTF2 nontarget RNAs) with hypo m^{6}A upon *CSTF2*-KD in PANC-1 cells with or without *CSTF2*-KD. **l** Shown are examples of transcripts experiencing m^{6}A level change and Pol II/Pol II-Ser2P binding densities change with or without *CSTF2* KD. CSTF2 binding in DNA and RNA were also revealed by CUT&Tag-seq and HITS-CLIP-seq, respectively. **m** Scheme of DRB/GRO-seq in this study. **n** Elongation rate of CSTF2 target RNAs was measured by DRB/GRO-seq in PANC-1 cells with or without *CSTF2* KD. *P* values were from two-sided student’s *t*-tests in **a**, from DRMEM motif analysis in **g**, **i**, and from Kolmogorov-Smirnov test in **m**, respectively.

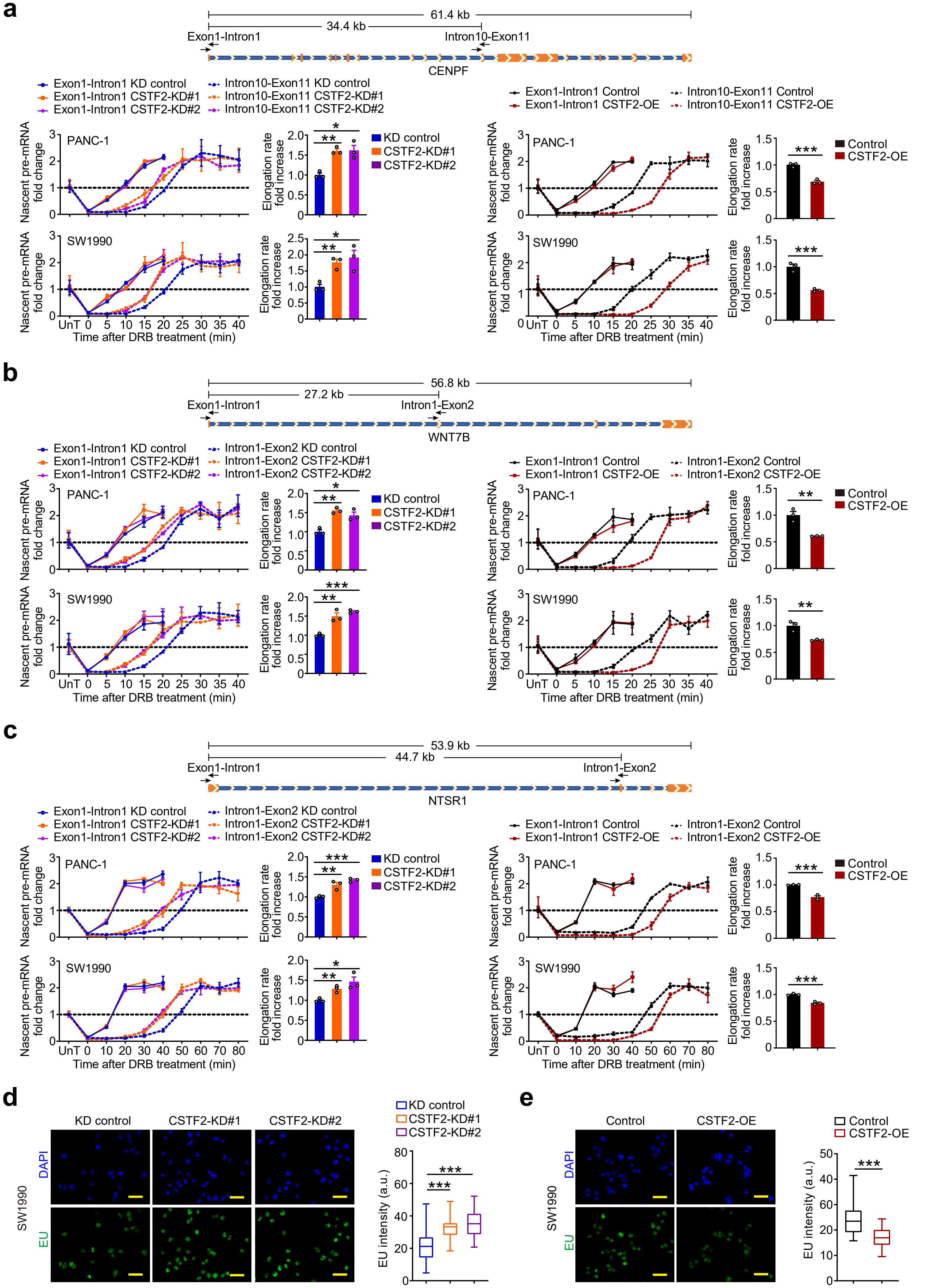
a
61.4 kb
34.4 kb
Exon1-Intron1
Intron10-Exon11
CENPF
Exon1-Intron1 KD control
Exon1-Intron1 CSTF2-KD#1
Exon1-Intron1 CSTF2-KD#2
Intron10-Exon11 KD control
Intron10-Exon11 CSTF2-KD#1
Intron10-Exon11 CSTF2-KD#2
Exon1-Intron1 Control
Exon1-Intron1 CSTF2-OE
Intron10-Exon11 Control
Intron10-Exon11 CSTF2-OE
PANC-1
SW1990
Nascent pre-mRNA fold change
Elongation rate fold increase
KD control
CSTF2-KD#1
CSTF2-KD#2
Control
CSTF2-OE
Time after DRB treatment (min)
UnT 0 5 10 15 20 25 30 35 40
b
56.8 kb
27.2 kb
Exon1-Intron1
Intron1-Exon2
WNT7B
Exon1-Intron1 KD control
Exon1-Intron1 CSTF2-KD#1
Exon1-Intron1 CSTF2-KD#2
Intron1-Exon2 KD control
Intron1-Exon2 CSTF2-KD#1
Intron1-Exon2 CSTF2-KD#2
Exon1-Intron1 Control
Exon1-Intron1 CSTF2-OE
Intron1-Exon2 Control
Intron1-Exon2 CSTF2-OE
c
53.9 kb
44.7 kb
Exon1-Intron1
Intron1-Exon2
NTSR1
UnT 0 10 20 30 40 50 60 70 80
d
KD control
CSTF2-KD#1
CSTF2-KD#2
SW1990
DAPI
EU
EU intensity (a.u.)
e
Control
CSTF2-OE

Supplementary Fig. 6. CSTF2 promotes mRNA-m^{6}A via slowing elongation.

a−c Diagram demonstrating the positions of PCR products in investigating the DRB-treated RNA Pol II elongation rate. Nascent mRNA production in two different regions of *CENPF* (**a**), *WNT7B* (**b**), *NTSR1* (**c**) after release from DRB inhibition in PANC-1 (*upper panel*) and SW1990 (*lower panel*) cells with *CSTF2* KD (*left panel*) or overexpression (*right panel*). The histograms show fold changes of elongation rates. Data in this figure are means ± S.E.M. (n = 3) from three independent experiments. **d, e** 5-ethynyluridine (EU) labeling affected by *CSTF2* KD (**d**) or overexpression (**e**) in SW1990 cells. Shown are representative images (*left panel*) of three independent experiments and quantification analysis of EU signals from one representative experiment (*right panel*). Scale bar, 100 μm. Boxplots indicate median (middle line), 25th, 75th percentile (box) and 5th and 95th percentile (whiskers) (Cell number: n = 33, 40 and 36 for KD control, CSTF2-KD#1 and CSTF2-KD#2; n = 38 and 27 for Control and CSTF2 OE, respectively). *, $P < 0.05$; **, $P < 0.01$ and ***, $P < 0.001$ of Student's *t*-test.

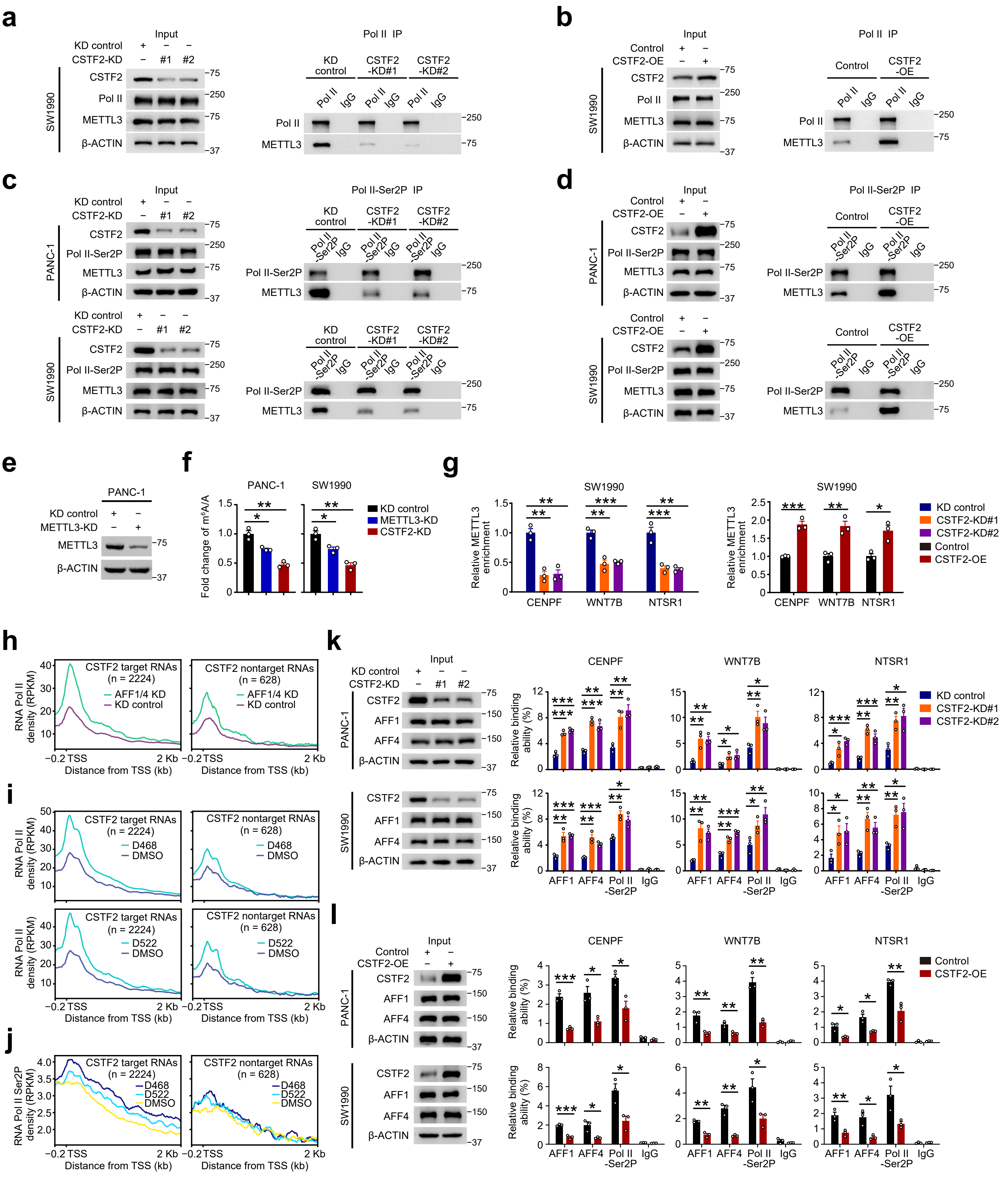
a
Input
KD control
CSTF2-KD
#1
#2
SW1990
CSTF2
Pol II
METTL3
β-ACTIN
Pol II IP
KD control
CSTF2 -KD#1
CSTF2 -KD#2
Pol II
IgG
METTL3
b
Input
Control
CSTF2-OE
Pol II IP
c
Input
Pol II-Ser2P IP
PANC-1
Pol II-Ser2P
d
e
PANC-1
METTL3-KD
f
PANC-1
SW1990
Fold change of m6A/A
KD control
METTL3-KD
CSTF2-KD
g
SW1990
Relative METTL3 enrichment
CENPF
WNT7B
NTSR1
KD control
CSTF2-KD#1
CSTF2-KD#2
Control
CSTF2-OE
h
CSTF2 target RNAs
(n = 2224)
CSTF2 nontarget RNAs
(n = 628)
AFF1/4 KD
KD control
RNA Pol II density (RPKM)
Distance from TSS (kb)
−0.2 TSS
2 Kb
i
D468
DMSO
D522
j
RNA Pol II Ser2P density (RPKM)
k
AFF1
AFF4
Relative binding ability (%)
Pol II -Ser2P
IgG
l

Supplementary Fig. 7. CSTF2-induced moderative elongation facilitates METTL3-catalyzed m^6A modifications.

a, b Western blotting analysis of the immunoprecipitated product by antibody against Pol II showing effects of *CSTF2* KD (**a**) or CSTF2 overexpression (**b**) on the interaction between Pol II and METTL3 in SW1990 cells. **c, d** Western blotting analysis of the immunoprecipitated product by antibody against Pol II Ser2P showing effects of *CSTF2* KD (**c**) or *CSTF2* overexpression (**d**) on the interaction between Pol II and METTL3 in PANC-1 (*upper*) and SW1990 (*lower*) cells. **e** Western blotting analysis of METTL3 in PANC-1 upon *METTL3* knockdown. **f** The effect of *CSTF2* or *METTL3* expression change on m^6A level in PANC-1 and SW1990 cells determined by m^6A-LC-MS. **g** CLIP-qPCR showed that *CSTF2* KD impaired (*left panel*) but CSTF2 overexpression enhanced (*right panel*) the binding ability of METTL3 to target transcripts in SW1990. **h, i** Effects of AFF1/4 knockdown (**h**) or AFF inhibitor (D468 and D522) treatment (**i**) on RNA Pol II density along the mRNA (*left*: CSTF2 target RNAs; *right*: CSTF2 nontarget RNAs) in HEK293T cells. **J** Effects of AFF inhibitor (D468 and D522) treatment on RNA Pol II-Ser2P density along the mRNA (*left*: CSTF2 target RNAs; *right*: CSTF2 nontarget RNAs) in HEK293T cells. **k, l** ChIP-qPCR showed that *CSTF2 KD* enhanced (**k**) but *CSTF2* overexpression impaired (**l**) the enrichment of AFF1, AFF, Pol II-Ser2P to target genes in PDAC cells. Data in **f**, **g**, **k**, **l** are means ± S.E.M. in (n = 3) independent experiments. *, $P < 0.05$; **, $P < 0.01$ ** and ***, $P < 0.001$ of Student's *t*-test compared with each control.

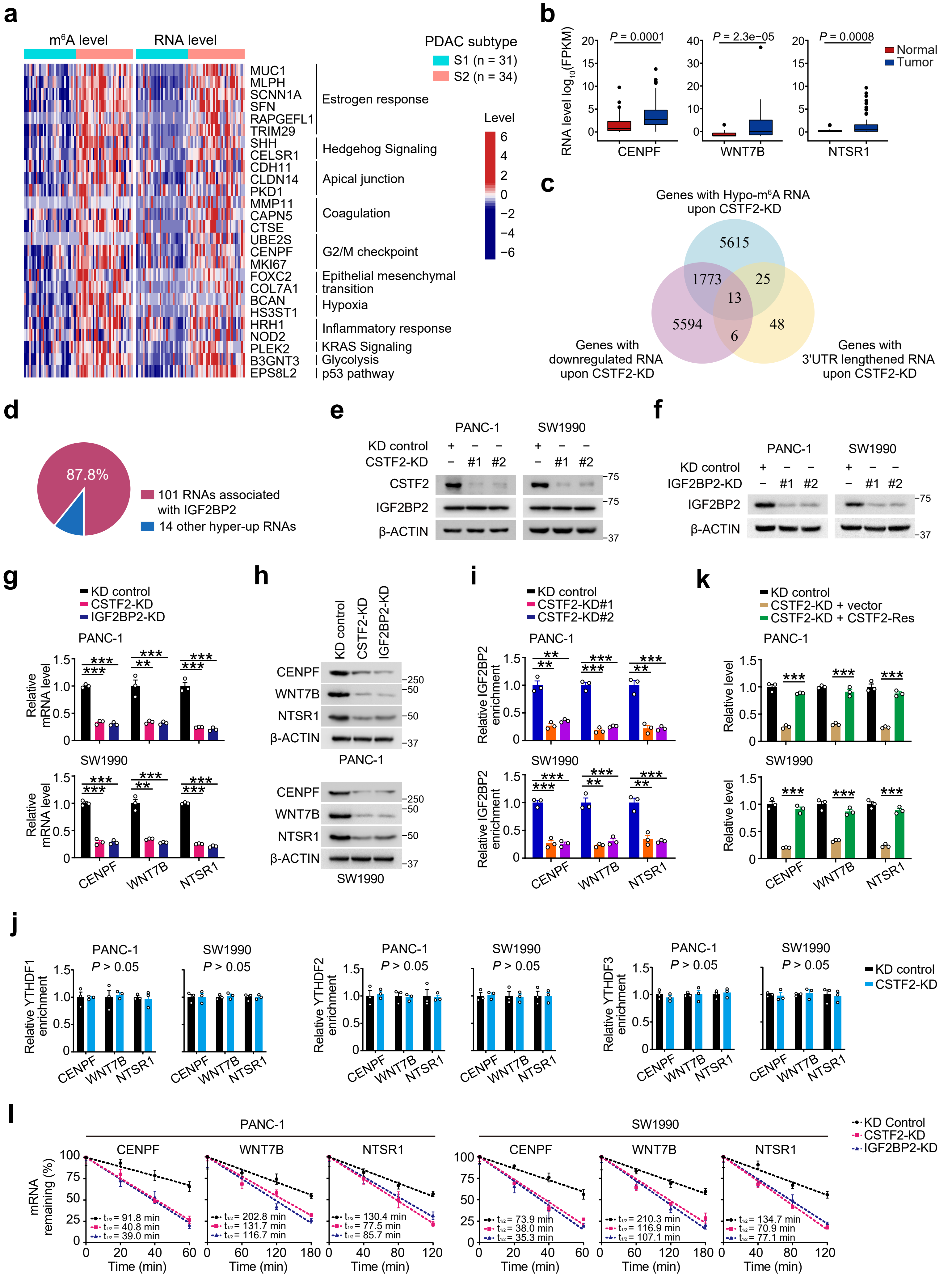

a
m6A level
RNA level
PDAC subtype
S1 (n = 31)
S2 (n = 34)
Level
MUC1
MLPH
SCNN1A
SFN
RAPGEFL1
TRIM29
Estrogen response
SHH
CELSR1
Hedgehog Signaling
CDH11
CLDN14
PKD1
Apical junction
MMP11
CAPN5
CTSE
Coagulation
UBE2S
CENPF
MKI67
G2/M checkpoint
FOXC2
COL7A1
Epithelial mesenchymal transition
BCAN
HS3ST1
Hypoxia
HRH1
NOD2
Inflammatory response
PLEK2
KRAS Signaling
B3GNT3
Glycolysis
EPS8L2
p53 pathway
b
RNA level log10(FPKM)
P = 0.0001
P = 2.3e−05
P = 0.0008
CENPF
WNT7B
NTSR1
Normal
Tumor
c
Genes with Hypo-m6A RNA upon CSTF2-KD
5615
1773
25
13
5594
48
6
Genes with downregulated RNA upon CSTF2-KD
Genes with 3'UTR lengthened RNA upon CSTF2-KD
d
87.8%
101 RNAs associated with IGF2BP2
14 other hyper-up RNAs
e
PANC-1
SW1990
KD control
CSTF2-KD
CSTF2
IGF2BP2
β-ACTIN
f
IGF2BP2-KD
g
KD control
CSTF2-KD
IGF2BP2-KD
Relative mRNA level
h
CENPF
WNT7B
NTSR1
i
CSTF2-KD#1
CSTF2-KD#2
Relative IGF2BP2 enrichment
k
CSTF2-KD + vector
CSTF2-KD + CSTF2-Res
Relative level
j
Relative YTHDF1 enrichment
Relative YTHDF2 enrichment
Relative YTHDF3 enrichment
P > 0.05
KD Control
l
mRNA remaining (%)
Time (min)
t1/2 = 91.8 min
t1/2 = 40.8 min
t1/2 = 39.0 min
t1/2 = 202.8 min
t1/2 = 131.7 min
t1/2 = 116.7 min
t1/2 = 130.4 min
t1/2 = 77.5 min
t1/2 = 85.7 min
t1/2 = 73.9 min
t1/2 = 38.0 min
t1/2 = 35.3 min
t1/2 = 210.3 min
t1/2 = 116.9 min
t1/2 = 107.1 min
t1/2 = 134.7 min
t1/2 = 70.9 min
t1/2 = 77.1 min

Supplementary Fig. 8. Hyper m^{6}A methylation enhances mRNA stability.

a Heatmap showing the differential m^{6}A and RNA level of indicated genes involved in distinct pathways between S2 (n = 34) and S1 (n = 31) PDAC tissues. **b** Box plot showing differential expression of indicated genes in PDAC tissues compared with matched normal tissues. Boxplots indicate median (middle line), 25th, 75th percentile (box) and 5th and 95th percentile (whiskers). *P* values were from Wilcoxon rank-sum test. **c** Venn plot of genes with hypo-methylated upon *CSTF2* KD, genes with lengthened 3'UTR upon *CSTF2* KD and genes with downregulation upon *CSTF2* KD. **d** Pie chart showing the percentage of IGF2BP2-positively correlated RNAs in all S2-hyper-up RNAs. **e** Western blotting analysis of CSTF2 and IGF2BP2 in PDAC cells upon *CSTF2* KD. **f** Western blotting analysis of IGF2BP2 in PDAC cells upon *IGF2BP2* KD. **g, h** Effect of *CSTF2* KD or *IGF2BP2* KD on RNA (**g**) and protein (**h**) level of *CENPF*, *WNT7B*, *NTSR1* determined by qRT-PCR and western blotting, respectively. **i** Relative binding ability of IGF2BP2 to indicated RNAs upon *CSTF2* KD determined by CLIP-qPCR. **j** CLIP-qPCR showed the binding ability of YTHDF1, YTHDF2, YTHDF3 to target transcripts in cells with or without *CSTF2* KD. **k** Relative RNA level of *CENPF*, *WNT7B*, *NTSR1* upon *CSTF2* KD with or without rescued with shRNA-resistant CSTF2 determined by qRT-PCR. **l** Half-lives of target transcript upon *CSTF2* KD or *IGF2BP2* KD determined by qRT-PCR. Data of **g**, **i−l** were mean ± S.E.M. in (n = 3) independent experiments. **, $P < 0.01$; ***, $P < 0.001$ of Student's *t*-test compared with each control.

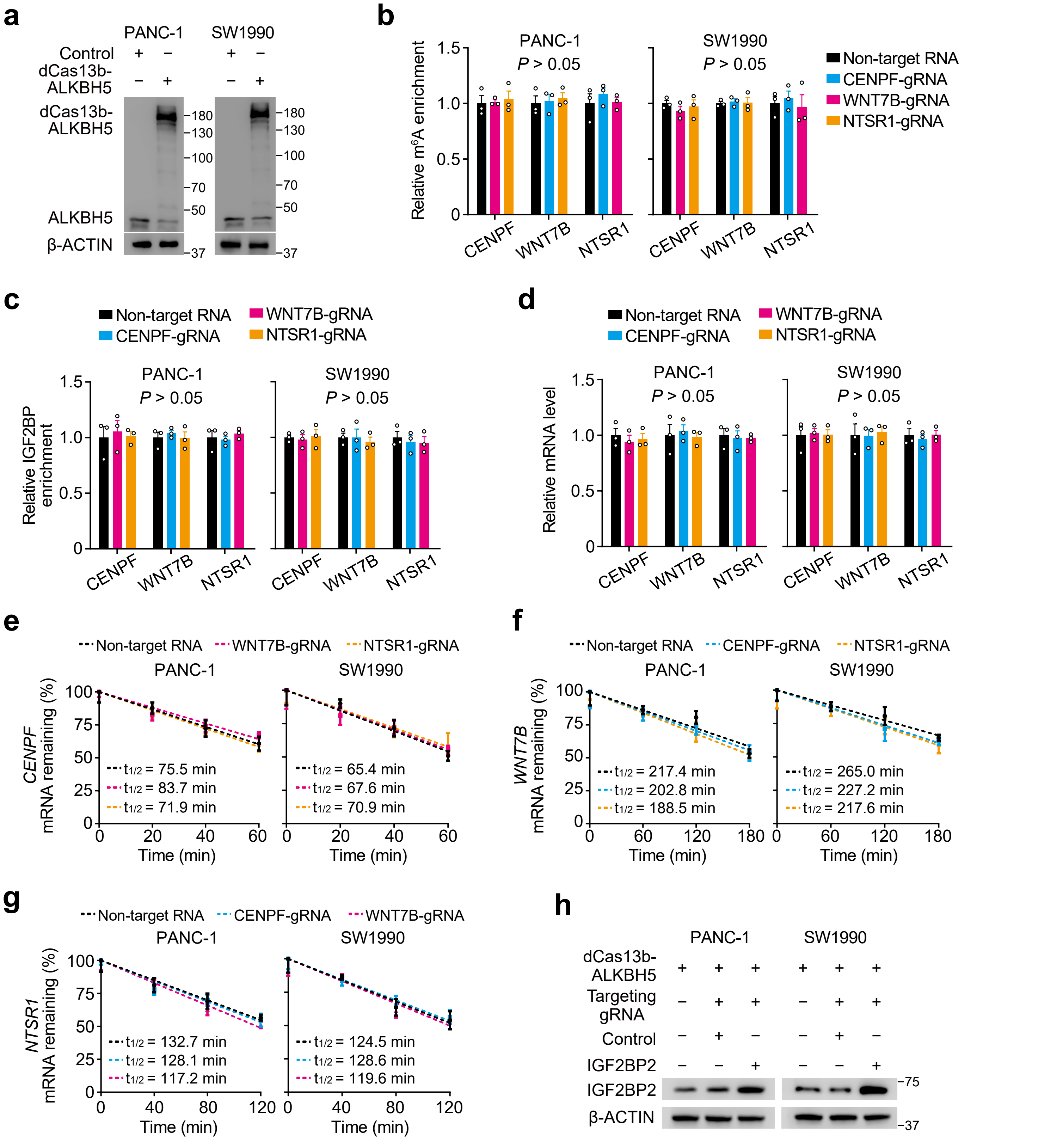

Supplementary Fig. 9. Effects of m⁶A level alteration by using the dCas13 based m⁶A editing system.
a Western blot analysis of dCas13 based m⁶A editing system. **b** Relative m⁶A enrichment of indicated transcript detected by MeRIP-qPCR upon transfected with dCas13b-ALKBH5 and non-target gRNA or gRNA targeting m⁶A of specific RNA, respectively. **c** Relative IGF2BP2 enrichment of indicated transcript detected by CLIP-qPCR upon transfected with dCas13b-ALKBH5 and non-target gRNA or gRNA targeting m⁶A of specific RNA, respectively. **d−g** Relative mRNA level **(d)** and half-lives **(e−g)** of *CENPF*, *WNT7B*, *NTSR1* detected by qRT-PCR upon transfected with dCas13b-ALKBH5 and non-target gRNA or gRNA targeting m⁶A of indicated RNA, respectively. **h** Western blot analysis of dCas13 based m⁶A editing system with or without forced expression of IGF2BP2. Data were mean ± S.E.M. of three independent experiments in **b−g.** *P* value were from Student's *t*-test compared with each control.

Supplementary Table 1. Baseline demographic and clinical characteristics of PDAC patients in this study

	PDAC S1 (n = 31)	PDAC S2 (n = 34)	*P* value[5]
**Age, mean (SEM[1])**	59.1 (2.1)	62.7 (2.3)	0.266
**Sex, *N* (%)**			0.184
Male	15 (48.4)	22 (64.7)	
Female	16 (51.6)	12 (35.3)	
**Smoking status[2], *N* (%)**			> 0.5
Smoker	9 (29.0)	11 (32.4)	
Nonsmoker	22 (71.0)	23 (67.6)	
**Drinking status[3], *N* (%)**			0.456
Drinker	5 (16.1)	8 (23.5)	
Nondrinker	26 (83.9)	26 (76.5)	
**Tumor differentiation, *N* (%)**			> 0.5
Well	6 (19.4)	7 (20.6)	
Moderate	20 (64.5)	20 (58.8)	
Poor	5 (16.1)	7 (20.6)	
**Neural invasion, *N* (%)**			0.039
Yes	7 (22.6)	16 (47.1)	
No	24 (77.4)	18 (52.9)	
**Vascular invasion, *N* (%)**			> 0.5
Yes	6 (19.4)	7 (20.6)	
No	25 (80.6)	27 (79.4)	
**Lymph node metastasis, *N* (%)**			0.375
Yes	13 (41.9)	18 (52.9)	
No	18 (58.1)	16 (47.1)	
**Tumor stage[4], *N* (%)**			0.264
I+ II	23 (74.2)	29 (85.3)	
III+ IV	8 (25.8)	5 (14.7)	
**Survival status, *N* (%)**			0.039
Alive	8 (25.8)	2 (5.9)	
Deceased	23 (74.2)	32 (94.1)	
**Stromal content, mean (SEM)**	31.8 (1.4)	30.7 (1.6)	> 0.5

[1]SEM, standard error of mean.

[2]Individuals who smoked an average of <1 cigarette/day and for <1 year in their lifetime were defined as nonsmokers; otherwise, they were defined as smokers.

[3]Individuals were classified as drinkers if they drank at least twice a week and continuously for at least 1 year during their lifetime; otherwise, they were defined as nondrinkers.

[4]Tumor TNM staging were reviewed by at least 3 pathologists and defined according to the American Joint Committee on Cancer (AJCC) 7th edition.

[5]*P* values were calculated by two-sided Student's *t*-tests (age and stromal content) or Fisher's exact test .

Supplementary Table 2. Validation results of selected differentially methylated m^{6}As between PDAC and normal tissues by MeRIP qRT-PCR method

m^{6}A[1]	Gene symbol	RNA type[2]	Region[3]	m^{6}A specific qRT-PCR						
				Upstream Primer Sequence (5'→3')	Downstream Primer Sequence (5'→3')	Average level in tumor	Average level in normal	Fold change (T/N)	*P* value[4]	Validated
chr1:156637890-156638313	RP11-284F21.9	LncRNA	exon	ATGAGCTGGTCAGACCGCGCTTTG	CCATAGGAGGCTGGGAGAGGCACT	0.81	0.15	5.33	3.88E-05	Yes
chr1:178865092-178865816	ANGPTL1	mRNA	CDS	AAGATACCCTCGTGCCACAGATGGT	TTCTGCCTGGAGAGCACATCCTT	1.26	2.33	0.54	8.34E-03	Yes
chr1:36319598-36321379	SH3D21	mRNA	3UTR	AGGTGATGCAGGGGACCCAGAA	TAGGCAGAGTTTACTTTCTCGCGTT	2.31	1.10	2.09	1.10E-03	Yes
chr11:618614-619205	CDHR5	mRNA	CDS	ACAACTCTGAGGCCACCAACCTCGT	TGGTTGGTGGGAGGTGCTGGTT	1.74	1.03	1.69	7.03E-03	Yes
chr11:77123734-77124356	CAPN5	mRNA	3UTR	TCAACTGTCACCACTGCTAAG	AGAACACCAGGTATTGCTGAT	1.72	1.15	1.50	5.57E-08	Yes
chr15:90919736-90920346	MAN2A2	mRNA	3UTR	TCTATTTGGAGCCCATGGAGAT	ATGAGGGCAGAGCCCAGTGT	1.28	1.86	0.69	4.39E-02	Yes
chr16:23690323-23691518	ERN2	mRNA	Stop codon	AGAAGCACCACTACAGGGAGC	AGTCTGGCGGGTAGTAGGGCAGGA	1.48	0.66	2.26	7.08E-06	Yes
chr16:2831942-2832405	ZG16B	mRNA	intron	AGCAAGGACCGCTATTTCTATT	AGTTTGCTGAGTATGTGAGATTAA	1.47	0.55	2.69	1.50E-06	Yes
chr16:68698181-68698864	CDH3	mRNA	3UTR	TTCAAGAAGCTGGCAGACATGTA	ACTTCCTGACAAGCTCCGAAG	1.51	0.79	1.90	2.87E-09	Yes
chr16:86581265-86581619	FOXL1	mRNA	3UTR	TGTTGGTCAATGTGTTGGAAAGGAC	AGCCTGGATCTCAGCAATGGCTCCT	1.45	0.77	1.88	4.49E-07	Yes
chr17:43861966-43862983	CD300LG	mRNA	3UTR	ATTCTGGCTTCTCTTTGAACCA	AATGCAGACTCTAAGCTGGAG	0.56	1.01	0.55	6.91E-03	Yes
chr17:80964686-80965088	RPTOR	mRNA	3UTR	AAGAGCGAGCGAGAGGCGCT	GCTTCCTGCTCAGTGATGCT	1.39	2.36	0.59	7.61E-03	Yes
chr19:41206396-41207418	CYP2S1	mRNA	3UTR	ACTGACCTTCACTCCACCACGCA	TCTTAGCAGCATGAGTCAAGTGA	1.32	0.70	1.90	5.11E-04	Yes
chr19:48702801-48704688	FUT2	mRNA	CDS	ATCTTCAGAATCACCCTGCCG	TGAACTCCTGGAGGATCTCCT	1.95	1.07	1.81	2.52E-10	Yes
chr19:49064299-49064759	CTB-60B18.23	mRNA	intron	TCTGAGGGAGAAGGGTGCTG	TCAGACCGAGTAGTGCAGAC	1.09	0.39	2.76	4.34E-03	Yes
chr2:5696221-5697163	SOX11	mRNA	3UTR	AAGGAGAGAAGTGGTCGGTGTCTGT	ACAGAGGCCATGCCATGTTCCT	0.74	0.35	2.12	3.39E-12	Yes
chr20:54028271-54028882	BCAS1	mRNA	CDS	AGGGATCCCACGCTTCTCCCACCT	ACTGCCCTGATAAGCCAGGAA	2.95	1.42	2.08	3.90E-16	Yes
chr20:63529193-63529722	PTK6	mRNA	3UTR	ATGTCCAACCATGAGGCCTTCCTGA	ATGCCCGCTCCACAGCAGCTCAGGT	1.25	0.64	1.95	3.30E-04	Yes
chr22:45921180-45921758	WNT7B	mRNA	3UTR	TATCAAGCAAATCAAAATATCAC	AAGTGGGACAAACATCCCTGA	1.45	0.29	4.97	9.63E-13	Yes
chr3:124927664-124928084	MUC13	mRNA	CDS	AGCTGATACCACTGAAACTAATT	AGGTAGCTAATGAATTTACAT	1.18	0.90	1.31	2.90E-02	Yes
chr3:195789583-195791565	MUC4	mRNA	CDS	AAGCTACAGTGTGACTCAGATGAT	TGAGTGTGACCCCTTTGGGAAA	1.13	0.47	2.41	2.75E-05	Yes
chr5:135031120-135031681	PITX1	mRNA	CDS	AGGGAAGACCGCCAGCGCTGGT	TGAAGTGCGTACGTTGCCGCCGCT	2.09	0.66	3.18	4.28E-13	Yes
chr5:160563837-160564139	ATP10B	mRNA	3UTR	TATCACTTGAGAACCTCCTCAGCAA	AGCTCCAGGACAGTGCTGCAAAT	1.16	0.62	1.86	1.50E-04	Yes
chr5:41312412-41312820	PLCXD3	mRNA	intron	TACAAGTGTGTTAAAATTGAA	AATTCCTTCCTTCCTTCTGTCT	1.97	2.84	0.69	8.70E-02	No
chr6:10397719-10398393	TFAP2A	mRNA	3UTR	GTGAAGCCAGCAGAGAAAAGTT	AACTGAAGTATGTAAGGGAAGGTGG	0.96	0.36	2.67	1.76E-07	Yes
chr6:30948675-30952367	DPCR1	mRNA	CDS	ATCTCTAGCAGAGCCTACAGAA	TCATTGGCTGTCCTTTCTCTATT	1.78	0.62	2.86	1.84E-09	Yes
chr7:29566178-29567256	PRR15	mRNA	Stop codon	AGCCCGGACTGGACCAGCAGCT	TCACCAGGAAAGCCTGCCTCCT	1.77	0.80	2.21	8.36E-10	Yes
chr8:101688671-101689421	NCALD	mRNA	intron	AGCAGTGCCGGCCAGTTCTGA	AGCTGAAGGCGTCACGGAGGAA	1.08	1.74	0.62	3.40E-04	Yes
chr8:143743217-143743756	FAM83H-AS1	mRNA	intron	TGCATCTTTGTTAACATTTAGACAT	TTAAATATGCACTGTGAGGCAGATG	1.09	0.46	2.39	1.15E-07	Yes

[1] m^{6}As are represented by human hg38 genomic positions.
[2] LncRNA, long non-coding RNA
[3] The regions in mRNA structure.
[4] Student's *t*-test.

Supplementary Table 3. The primers sequences used in this study

Primers for quantitative real time-PCR		
Gene symbol	Forward primer (5'→3')	Reverse primer (5'→3')
CSTF2	AGCACTTCGAGTGGACAATGC	AAGGTGACTCAATGACAGGGG
METTL3	TTGTCTCCAACCTTCCGTAGT	CCAGATCAGAGAGGTGGTGTAG
METTL14	GAGTGTGTTTACGAAAATGGGGT	CCGTCTGTGCTACGCTTCA
WTAP	TTGTAATGCGACTAGCAACCAA	GCTGGGTCTACCATTGTTGATCT
FTO	AACACCAGGCTCTTTACGGTC	TGTCCGTTGTAGGATGAACCC
ALKBH5	CGGCGAAGGCTACACTTACG	CCACCAGCTTTTGGATCACCA
RBM15	GTGAGGACTCGACTTCCCG	GCCGCTATCGGTCTTTCCG
RBM15B	TACACGGAGGCTACCAGTACA	GTCGTACAGCCCGTAGTAGTC
IGF2BP2	TCGAGACCCTCTCGGGTAAA	GTTGACAACGGCGGTTTCTG
CENPF	GGGAGAATGATTCACTTA	TCGGCATCAAGAATCACT
WNT7B	GAGGATTCTCGGCACTAACA	GGAAAGGAAACAGAGGGTGT
NTSR1	AGGACACCACATGGGAGGG	CACAGCCACAAGCAAAGCA
U2AF2	TCTTCAACGCCCAGATGC	CGTTCAGGTAGTTGGGTAAGC
CAPRIN1	AACGGTTGAGGTGGTAAAT	GCAGATACAATGGCAGGAT
BUD13	TCTGAAGCGTTACTTGTCC	ATGTCTGTTTGAGGGTAGGT
β-ACTIN	CAGGGCGTGATGGTGGGCATG	GTAGAAGGTGTGGTGCCAGATT
Primers for MeRIP-qPCR, CLIP-qPCR and ChIP-qPCR		
CENPF-m^{6}A	GGAGCTAGTGATTCTTGA	TCTGAAGCATCTCCACTG
WNT7B-m^{6}A	CCAGGGGTCTTGGTGCTC	GCCTGGGAACTGGTCTGG
NTSR1-m^{6}A	GTGCTTTGCTTGTGGCTGTG	CCTGGGACCTGGCATCTTAC
Primers for DRB-qPCR		
CENPF Exon1-Intron1	CGAGAGGTCGTTTTCCCGT	GGGGCGGATTCTCCAGTTC
CENPF Exon10-Intron11	ACTTGAGCATTCGTTTTGGCT	CAGGTGGCAGACTTCTCTGG
WNT7B Exon1-Intron1	TTTCTCTGCTTTGGCGTCCT	CAGCTCCTTCGTGCTGTCTT
WNT7B Intron1-Exon2	GTGACTGAGAGGCTGAGTGG	GGCCAGGAATCTTGTTGCAG
NTSR1 Exon1-Intron1	TATGCTGTTCACCATGGGCG	TCCCACTCCCTACCCAGAAG
NTSR1 Intron1-Exon2	CAGCTGAGGGTGCATGAGTC	AGCTTGTTGGCGATGATGGT

Supplementary Table 4. The RNA knockdown and gRNA sequences used in this study

	RNA knockdown sequences (5'→3')
KD Control	UUCUCCGAACGUGUCACGUTT
CSTF2-KD#1	GACCAAGAGACAGCACUUATT
CSTF2-KD#2	GGGAGGCACUUUACUUUCUTT
U2AF2-KD#1	GCAGUACAAGGCCAUGCAATT
U2AF2-KD#2	CCAACUACCUGAACGAUGATT
CAPRIN1-KD#1	GGAGCAGCUUAUGAGAGAATT
CAPRIN1-KD#2	CCAUGCAAACGGUGUUCAATT
BUD13-KD#1	CUGCCAGAAAGCAACUGAUTT
BUD13-KD#2	GCUCAAGGAACAGGAUCAATT
CENPF-KD#1	GACCCAGAAACUAGCUUAUTT
CENPF-KD#2	CCAGCUUCGUGGAGAAUUATT
WNT7B-KD#1	GCAGGGCUACUACAACCAATT
WNT7B-KD#2	CCACCUUCCUGCGCAUCAATT
NTSR1-KD#1	GCACAUUCAGCAUGGCCAUTT
NTSR1-KD#2	GCCUCAUGUUCUGCUACAUTT
METTL3-KD#1	GGAGATCCTAGAGCTATTA
METTL3-KD#2	GCACATCCTACTCTTGTAA
IGF2BP2-KD#1	CAGUUUGAGAACUACUCCUTT
IGF2BP2-KD#2	GAAACAGGGACCAAGAUAATT
	gRNA sequence for dCas13b-ALKBH5 system (5'→3')
Non-Target-gRNA	GTAATGCCTGGCTTGTCGACGCATAGTCTG
CENPF-m^6A-gRNA	CAGACCTTAACGTGACAAGGTCTAATTCAAAAACTTTCAG
WNT7B-m^6A-gRNA	CCCAGAGAGAAGAAAGAGAACTTGCCGGGCCCCGTCGGGG
NTSR1-m^6A-gRNA	CTCCAGACCTCAGCCTGCACAGCCACAAGCAAAGCACACG
