# Supplementary material for: CSTF2 mediated mRNA N6-methyladenosine modification drives pancreatic ductal adenocarcinoma m6A subtypes
Source: Nat Commun. 2023 Oct 10;14:6334. doi: 10.1038/s41467-023-41861-y (PMC10564946; doi:10.1038/s41467-023-41861-y)
Supplement: Supplementary file 2 — Description of Additional Supplementary Files [file 41467_2023_41861_MOESM2_ESM.pdf]

Description of Additional Supplementary Files

Supplementary Data 1: Data quality information for m^6A-seq in tissues.

Supplementary Data 2: Total m^6As identified by m^6A sequencing in 65 PDAC tumor and 33 normal tissue samples.

Supplementary Data 3: Differentially methylated m^6As between PDAC and adjacent normal tissues.

Supplementary Data 4: Differentially expressed aberrant m^6As between S2 and S1 PDAC tissues.
