# Supplementary material for: CSTF2 mediated mRNA N6-methyladenosine modification drives pancreatic ductal adenocarcinoma m6A subtypes
Source: Nat Commun. 2023 Oct 10;14:6334. doi: 10.1038/s41467-023-41861-y (PMC10564946; doi:10.1038/s41467-023-41861-y)
Supplement: Supplementary file 7 — Reporting Summary [file 41467_2023_41861_MOESM7_ESM.pdf]

nature portfolio

Corresponding author(s): Zhixiang Zuo

Last updated by author(s): Aug 29, 2023

Reporting Summary

Nature Portfolio wishes to improve the reproducibility of the work that we publish. This form provides structure for consistency and transparency in reporting. For further information on Nature Portfolio policies, see our Editorial Policies and the Editorial Policy Checklist.

Statistics

For all statistical analyses, confirm that the following items are present in the figure legend, table legend, main text, or Methods section.

n/a	Confirmed	
☐	☒	The exact sample size (*n*) for each experimental group/condition, given as a discrete number and unit of measurement
☐	☒	A statement on whether measurements were taken from distinct samples or whether the same sample was measured repeatedly
☐	☒	The statistical test(s) used AND whether they are one- or two-sided<br>*Only common tests should be described solely by name; describe more complex techniques in the Methods section.*
☐	☒	A description of all covariates tested
☐	☒	A description of any assumptions or corrections, such as tests of normality and adjustment for multiple comparisons
☐	☒	A full description of the statistical parameters including central tendency (e.g. means) or other basic estimates (e.g. regression coefficient) AND variation (e.g. standard deviation) or associated estimates of uncertainty (e.g. confidence intervals)
☐	☒	For null hypothesis testing, the test statistic (e.g. *F*, *t*, *r*) with confidence intervals, effect sizes, degrees of freedom and *P* value noted<br>*Give P values as exact values whenever suitable.*
☒	☐	For Bayesian analysis, information on the choice of priors and Markov chain Monte Carlo settings
☒	☐	For hierarchical and complex designs, identification of the appropriate level for tests and full reporting of outcomes
☐	☒	Estimates of effect sizes (e.g. Cohen's *d*, Pearson's *r*), indicating how they were calculated

Our web collection on statistics for biologists contains articles on many of the points above.

Software and code

Policy information about availability of computer code

Data collection	Public CLIP-seq data of m6A and CSTF2 are accessible under GEO number GSE147440 and GSE37398, respectively. All public sequencing data were downloaded using Wget 1.14, with code 'wget -c ftp://ftp.ncbi.nlm.nih.gov/sra/sra-instant/reads/ByRun/sra/SRR/*/*/*.sra'. Other data were generated using Illumina Novaseq 6000,Illumina HiSeqX Ten,Illumina HiSeq2500 or Illumina HiSeq4000.
Data analysis	STAR (v2.7.1a), FASTX-Toolkit (v0.0.14), MACS2 (v2.1.2), MeTPeak (v1.1), BEDTools (v2.30.0), Homer2 (v2.8), Gencode (version 44), RMBase (v3.0), MEME (v5.5.2), RSEM(v1.1.17), DESeq2 (v1.32.0), edgeR (v.3.34.1), ConsensusClusterPlus (v3.17), ClusterProfiler (v3.17), Cutadapt (v3.4), pyCRAC (v1.4), BWA (v0.7.15), CIMS, Survival, POSTAR2, limma (v3.48.3), VennDiagram (v1.6.0), survminer (v0.4.9), reshape2 (v1.4.4), Hmisc (v3.2.0), caret (v3.9.2), papeR (v1.19.3), ggforce (v0.4.1), data.table (v1.13.5), ggplot2 (v3.0.0), plyr (v1.8.8), ggrepel (v0.9.0), RcolorBrewer (v1.1-3), latticeExtra (v0.6-25), mediation (v4.5.0), randomForest (v4.7-1.1), GTEx, GEPIA (2.0), cBioPortal, CTK Documentation (v1.1.3), circos (v0.64), IGV (v2.3.93), GSEA (v4.1.0, for Gene Set Enrichment Analysis), Microsoft Excel 2016, Microsoft Word 2016, ImageJ (v1.46m), GraphPad Prism (version 8), SPSS software (version 20.0; IBM SPSS), RTCA software (v2.0), R (3.6.1) software for statistical computing. Custom codes developed for data preprocessing, analysis and visualization are available at GitHub https://github.com/canceromics/CSTF2_m6Asubtype_code.

For manuscripts utilizing custom algorithms or software that are central to the research but not yet described in published literature, software must be made available to editors and reviewers. We strongly encourage code deposition in a community repository (e.g. GitHub). See the Nature Portfolio guidelines for submitting code & software for further information.

Data

Policy information about availability of data

All manuscripts must include a data availability statement. This statement should provide the following information, where applicable:

- Accession codes, unique identifiers, or web links for publicly available datasets
- A description of any restrictions on data availability
- For clinical datasets or third party data, please ensure that the statement adheres to our policy

The raw sequence data reported in this paper have been deposited in the Genome Sequence Archive in BIG Data Center, Beijing Institute of Genomics (BIG), Chinese Academy of Sciences, under accession numbers HRA000095, HRA001663, HRA003601 and HRA004744 that are publicly accessible at https://bigd.big.ac.cn/gsa. Public CLIP-seq data of m6A and CSTF2 are accessible under GEO number GSE147440 and GSE37398, respectively. All custom code used to generate the data in this study is available upon reasonable request. Unprocessed blot of Figs. 5l, m and Supplementary Figs. 3c, g, h, j–m, o, 4a, c, d, 5b–e, 7a–e, h, i, 8e, f, h, 9a and h are available in Supplementary Figs. 10–13. The Source Data underlying Figs. 4b–f, 5j, k, o, p, 6d–i and Supplementary Figs. 3b, e, f, i–n, p, q, 4a,b, e–h, 5a, 6a–e, 7f, g, k, l, 8g, i–l, 9b–g are provided as a Source Data file.

Human research participants

Policy information about studies involving human research participants and Sex and Gender in Research.

Reporting on sex and gender	This study includes 65 patients with PDAC consists of 37 males and 28 females. For greater security and privacy, we decided not to provide gender information in each case.
Population characteristics	65 patients with PDAC were recruited and underwent pancreatectomy and received no treatment before surgery. The distributions of select characteristics are shown in Supplementary Table 1.
Recruitment	All patients were recruited at Sun Yat-sen University Sun Yat-sen Memorial Hospital (Guangzhou, China) between 2010 and 2018. The diagnosis of PDAC was histopathologically confirmed and tumor stage was classified according to the 7th edition of AJCC Cancer Staging System.
Ethics oversight	This study was performed according to the Declaration of Helsinki and approved by the Institutional Review Board of Sun Yat-sen University.

Note that full information on the approval of the study protocol must also be provided in the manuscript.

Field-specific reporting

Please select the one below that is the best fit for your research. If you are not sure, read the appropriate sections before making your selection.

[x] Life sciences [ ] Behavioural & social sciences [ ] Ecological, evolutionary & environmental sciences

For a reference copy of the document with all sections, see nature.com/documents/nr-reporting-summary-flat.pdf

Life sciences study design

All studies must disclose on these points even when the disclosure is negative.

Sample size	We performed m6A-seq on total RNAs of 98 surgically removed samples from 65 individuals with PDAC, including 33 pairs of tumor and their corresponding normal tissue samples and another 32 tumor samples. Besides, 18 cell line samples for m6A-seq and CLIP-seq. No statistical method was used to predetermine sample size. The size of each animal cohort was determined by estimating biologically relevant effect sizes between control and treated groups and then using the minimum number of animals that could reveal statistical significance using the indicated tests of significance. For in vitro experiments, n>3 were used, as a minimum, to allow for statistical analysis.
Data exclusions	No data were excluded from analysis.
Replication	In vitro experiments described were repeated with at least 2 independent cell lines and/or at least 2 separate occasions with similar results as those represented in the figures. In vivo experiments were conducted once. Animals were enrolled into in vivo experiments as they became available until sufficient numbers were obtained to determine experimental outcomes. Statistics and Reproducibility information can be found in the figures legends and/or Methods section.
Randomization	This study included 65 patients with PDAC recruited at Sun Yat-sen University Sun Yat-sen Memorial Hospital (Guangzhou, China) between 2010 and 2018. All patients underwent pancreatectomy and received no chemotherapy or radiotherapy before surgery. Allocation of mice into experimental groups was random. Randomization was not required for in vitro studies given that proper controls were included so all cell lines would receive the same treatment, regardless of subtype.
Blinding	Investigators were blinded to sample identity and group for histopathological assessments. For other analysis, authors were not blinded since no subjective analysis was performed that would be biased by the investigator knowing the sample identity. Objective data analysis was

measured using software listed above.

Reporting for specific materials, systems and methods

We require information from authors about some types of materials, experimental systems and methods used in many studies. Here, indicate whether each material, system or method listed is relevant to your study. If you are not sure if a list item applies to your research, read the appropriate section before selecting a response.

Materials & experimental systems

n/a	Involved in the study
☐	☒ Antibodies
☐	☒ Eukaryotic cell lines
☒	☐ Palaeontology and archaeology
☐	☒ Animals and other organisms
☒	☐ Clinical data
☒	☐ Dual use research of concern

Methods

n/a	Involved in the study
☒	☐ ChIP-seq
☒	☐ Flow cytometry
☒	☐ MRI-based neuroimaging

Antibodies

Antibodies used

Rabbit anti-m6A antibody (10 ug for MeRIP, 10 ug for miCLIP, Supplier: Synaptic Systems; Cat.: 202003)
Rabbit anti-CSTF2 antibody (WB: dil. 1:2000, 15 ug for CLIP, CUT&Tag:dil.1:50, IHC:dil.1:200 Supplier: Abcam; Cat.: ab200837)
Rabbit anti-CSTF2T antibody (WB: dil. 1:2000, Supplier: Abcam; Cat.:ab138486)
Rabbit anti-METTL3 antibody (WB: dil. 1:2000, Supplier: Abcam; Cat.: ab195352)
Rabbit anti-METTL14 antibody (WB: dil. 1:2000, Supplier: Abcam; Cat.: ab252562)
Rabbit anti-WTAP antibody (WB: dil. 1:1000, Supplier: Abcam; Cat.: ab195380)
Rabbit anti-FTO antibody (WB: dil. 1:1000, Supplier: Abcam; Cat.: ab126605)
Rabbit anti-ALKBH5 antibody (WB: dil. 1:2000, Supplier: Abcam; Cat.: ab195377)
Rabbit anti-WNT7B antibody (WB: dil. 1:2000, Supplier: Abcam; Cat.: ab227607)
Rabbit anti-IGF2BP2 antibody (WB: dil. 1:2000, 15 ug for CLIP, Supplier: Abcam; Cat.: ab128175)
Rabbit anti-beta-ACTIN antibody (WB: dil. 1:10000, Supplier: Abcam; Cat.: ab8227)
Mouse anti-RNA polymerase II C-terminal domain (CTD) antibody (WB: dil. 1:4000,5 ug for IP,CUT&Tag:dil.1:50, Supplier: Millipore; Cat.:#05-623)
Rabbit anti-RNA polymerase II C-terminal domain (CTD) Ser2 antibody (WB:dil. 1:2000, Supplier: Abcam; Cat.: ab193468)
Rabbit anti-RNA polymerase II C-terminal domain (CTD) Ser2 antibody (CUT&Tag:dil.1:50, Supplier: Active Motif; Cat.: 61083)
Mouse anti-RNA polymerase II C-terminal domain (CTD) Ser5 antibody (CUT&Tag:dil.1:50, Supplier: Invitrogen; Cat.: MA1-46093)
Rabbit anti-H3K36me3 antibody (CUT&Tag:dil.1:50, Supplier: Abcam; Cat.: ab9050)
Rabbit anti-H3K79me2 antibody (CUT&Tag:dil.1:50, Supplier: Abcam; Cat.: ab3594)
Rabbit anti-U2AF2 antibody (WB: dil.1:1000, Supplier: Proteintech; Cat.: 68166-1-lg)
Rabbit anti-CAPRIN1 antibody (WB: dil.1:1000, Supplier: Proteintech; Cat.: 15112-1-AP)
Rabbit anti-RBM15 antibody (WB: dil.1:2000, Supplier: Proteintech; Cat.: 10587-1-AP)
Rabbit anti-RBM15B antibody (WB: dil.1:2000, Supplier: Proteintech; Cat.: 67506-1-lg)
Rabbit anti-Lamin B1 antibody (WB: dil.1:4000, Supplier: Proteintech; Cat.: 12987-1-AP)
Mouse anti-GAPDH antibody (WB: dil.1:8000, Supplier: Proteintech; Cat.: 60004-1-lg)
Rabbit anti-AFF4 antibody (WB: dil.1:1000, Supplier: Proteintech; Cat.: 14662-1-AP)
Rabbit anti-CENPF antibody (WB: dil.1:2000, Supplier: Proteintech; Cat.: 28568-1-AP)
Rabbit anti-BUD13 antibody (WB: dil.1:2000, Supplier: Invitrogen; Cat.: A303-321A-1)
Rabbit anti-AFF1 antibody (WB: dil.1:1000, Supplier: Invitrogen; Cat.: A302-345A-1)
Mouse anti-NTSR1 antibody (WB: dil.1:1000, Supplier: Santa Cruz Bio; Cat.: sc-374492)
Mouse anti-Br-UTP antibody (GRO-seq: 5 ug, Supplier: Santa Cruz Bio; Cat.: sc-32323 AC)

Validation

All antibodies were used in accordance to the manufacturer guidelines and have been well documented in the literature. When possible, antibodies were validated beyond the manufacturers data sheet specification by blots (western) with known deficiencies for expression of the antigen.

Eukaryotic cell lines

Policy information about cell lines and Sex and Gender in Research

Cell line source(s)

Human PDAC cell lines PANC-1 and SW1990 and embryonic kidney cells 293T were purchased from the Cell Bank of Type Culture Collection of the Chinese Academy of Sciences Shanghai Institute of Biochemistry and Cell Biology.

Authentication

All cell lines are commercial and authenticated using the STR profiling.

Mycoplasma contamination

All cell lines were tested to be mycoplasma negative using the MycoBlue Mycoplasma Detector (Supplier: Vazyme; Cat.: D101-01)

Commonly misidentified lines (See ICLAC register)	No cell lines used in this study were found in the database of commonly misidentified cell lines that is maintained by ICLAC and NCBI Biosample.

Animals and other research organisms

Policy information about studies involving animals; ARRIVE guidelines recommended for reporting animal research, and Sex and Gender in Research

Laboratory animals	Aged 4–5 weeks' BALB/c nude mice mice were purchased from the Beijing Vital River Laboratory Animal Technology. All mice were maintained in our animal facility under controlled environmental conditions: 12/12 light/dark cycle, ambient temperature 20–25°C.
Wild animals	No wild animals was involved in this study.
Reporting on sex	Aged 4–5 weeks' female BALB/c nude mice were used in this study, and this is stated in the manuscript.
Field-collected samples	No field-collected samples was involved in this study.
Ethics oversight	All the animal experiments were approved by the Institutional Animal Care and Use Committee of Sun Yat-sen University Cancer Center, and the animals were handled in accordance with institutional guidelines.

Note that full information on the approval of the study protocol must also be provided in the manuscript.
